# Atomic Cu Sites Engineering Enables Efficient CO_2_ Electroreduction to Methane with High CH_4_/C_2_H_4_ Ratio

**DOI:** 10.1007/s40820-023-01188-1

**Published:** 2023-10-26

**Authors:** Minhan Li, Fangzhou Zhang, Min Kuang, Yuanyuan Ma, Ting Liao, Ziqi Sun, Wei Luo, Wan Jiang, Jianping Yang

**Affiliations:** 1 Institute of Functional Materials, State Key Laboratory for Modification of Chemical Fibers and Polymer Materials, College of Materials Science and Engineering, Donghua University, Shanghai, 201620 People’s Republic of China; 2 College of Materials Science and Engineering, Zhengzhou University, Zhengzhou, 450001 People’s Republic of China; 3 School of Mechanical, Medical and Process Engineering, School of Chemistry and Physics, Queensland University of Technology, 2 George Street, Brisbane, QLD 4001 Australia

**Keywords:** CO_2_RR, Cu single-atom catalyst, g-C_3_N_4_, Methane, CH_4_/C_2_H_4_ ratio

## Abstract

Electrochemical reduction of CO_2_ into high-value hydrocarbons and alcohols by using Cu-based catalysts is a promising and attractive technology for CO_2_ capture and utilization, resulting from their high catalytic activity and selectivity. The mobility and accessibility of active sites in Cu-based catalysts significantly hinder the development of efficient Cu-based catalysts for CO_2_ electrochemical reduction reaction (CO_2_RR). Herein, a facile and effective strategy is developed to engineer accessible and structural stable Cu sites by incorporating single atomic Cu into the nitrogen cavities of the host graphitic carbon nitride (g-C_3_N_4_) as the active sites for CO_2_-to-CH_4_ conversion in CO_2_RR. By regulating the coordination and density of Cu sites in g-C_3_N_4_, an optimal catalyst corresponding to a one Cu atom in one nitrogen cavity reaches the highest CH_4_ Faraday efficiency of 49.04% and produces the products with a high CH_4_/C_2_H_4_ ratio over 9. This work provides the first experimental study on g-C_3_N_4_-supported single Cu atom catalyst for efficient CH_4_ production from CO_2_RR and suggests a principle in designing highly stable and selective high-efficiency Cu-based catalysts for CO_2_RR by engineering Cu active sites in 2D materials with porous crystal structures.

**Electronic supplementary material:**

The online version of this article (10.1007/s40820-023-01188-1) contains supplementary material, which is available to authorized users.

## Highlights

- The Cu-doped graphitic carbon nitride (g-C_3_N_4_) material is synthesized by an in situ thermal polymerization strategy, through which the atomic dispersion and coordination structure of Cu on g-C_3_N_4_ are realized by regulating the doping level of Cu.
- High Faraday efficiency of CH_4_ of 49.04% and a maximum CH_4_/C_2_H_4_ ratio up to 35.03 are achieved for the first time on g-C_3_N_4_-supported Cu single-atom catalyst.
- Structure–activity relationship analysis based on experimental and theoretical studies demonstrates the well-defined Cu single atoms coordinated with N atoms in the nitrogen cavity of g-C_3_N_4_ are active sites for CO_2_-to-CH_4_.

**Electronic supplementary material:**

The online version of this article (10.1007/s40820-023-01188-1) contains supplementary material, which is available to authorized users.

## Graphical abstract

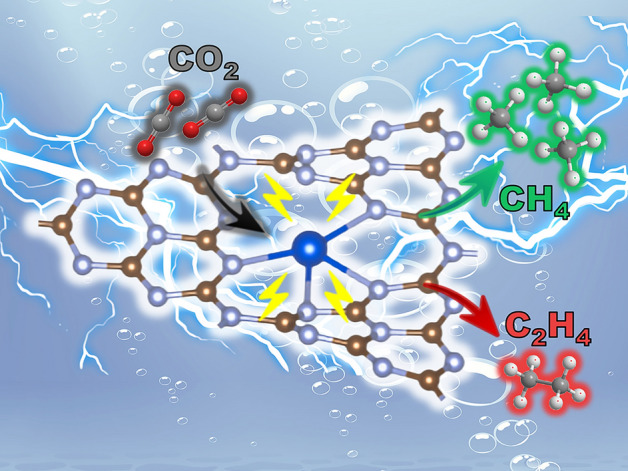

## Introduction

Cu-based electrocatalysts have received extensive attentions for CO_2_ electrochemical reduction reaction (CO_2_RR), because of their unique ability to convert CO_2_ into high-value deep reduction products [1–5]. Motivated by the great CO_2_-to-CO performance in CO_2_RR obtained on various carbon-supported single-atom catalysts, such as Ni [6–8], Fe [9, 10], Co [11, 12], and Zn [13], the Cu single-atom catalysts (Cu-SACs) have also been investigated for CO_2_RR [14]. Similar to the nanostructured Cu-based materials [15], the reported Cu-SACs also exhibited selectivity characteristics toward various reduction products in CO_2_RR, including CO [16], CH_4_ [17, 18], CH_3_OH [19, 20], C_2_H_5_OH [21, 22], and CH_3_COCH_3_ (acetone) [23]. The isolated Cu single atoms are usually regard as the main active centers for CO_2_RR, and the coordination environment and density of the atomic Cu species play a key role in the selectivity of the Cu-SACs. Cai and co-workers reported that carbon dots-supported Cu-SACs with unique Cu-N_2_O_2_ sites enabled efficient CO_2_RR to CH_4_ [24]. Xu and co-workers found that O atom coordinated single Cu atoms supported on carbon underwent a reversible transition between atomic sites and Cu_n_ clusters under CO_2_RR condition, and the later catalyzed CO_2_ into ethanol [21]. Zheng and co-workers reported that the distance between the atomic Cu-N_x_ sites was critical for the selectivity between CH_4_ and C_2_H_4_, where the neighboring Cu − N_x_ sites promoted the C–C coupling and decreased the CH_4_/C_2_H_4_ ratio [25]. During the synthesis of Cu-SACs, however, a high-temperature pyrolysis process is often involved, resulting in poorly defined coordination structure and the aggregation of the atomic Cu sites and thus poorer catalytic selectivity and less accessibility of the active sites [15, 26]. Compared to the pyrolytic SACs, molecular catalysts with explicit and tunable structures are more preferred, due to their well-defined and uniform coordination environments [24].

The challenges in preparing more structural stable and well-configured Cu-SACs triggered our interest in searching more suitable catalyst supports to anchor the active single atoms in a stable and well-coordinated environment. Similar to the well-documented graphene single-atom support, graphitic carbon nitride (g-C_3_N_4_) has a perfect 2D morphology with single atomic thickness to ensure the best accessibility of the active sites. But differently, g-C_3_N_4_ contains periodic heptazine units and well-defined “nitrogen pots” that consist of six lone pairs from pyridine-like nitrogen atoms among the heptazine units (nitrogen cavities) [27, 28]. The abundant and periodic nitrogen cavities in the g-C_3_N_4_ framework makes them become perfect anchoring points for incorporating dense metal atoms with defined distance and coordination numbers [29–31]. Various metal-doped g-C_3_N_4_ catalysts have been investigated for different electrocatalysis processes, such as hydrogen evolution reaction (HER) [32], oxygen evolution and reduction reaction (OER and ORR) [27, 33], nitrogen reduction reaction [34], and even CO_2_RR [35]. Specifically, it has been reported that a Cu-C_3_N_4_ catalyst exhibited strong CO_2_ affinity for promoted CO_2_ adsorption toward deep reduction for converting CO_2_ into high-value hydrocarbons. However, the Faraday efficiencies (FEs) of these deep reduction reactions were very low, and the role of the Cu sites incorporated in g-C_3_N_4_ framework for CO_2_RR remains unclear [36]. Therefore, further efforts in further enhancing the FEs of the Cu-g-C_3_N_4_ catalytic system together with a clear mechanism understanding are urgently needed.

In this work, a facile thermal polymerization method is employed to synthesize Cu single atoms loaded in g-C_3_N_4_ catalysts with different site distance and coordination environments (denoted as Cu_x_-CN) for CO_2_RR by adjusting the ratios of Cu to g-C_3_N_4_ in the precursors during the preparation. The synthesis–structure–activity analysis of Cu_x_-CN catalysts demonstrated that the Cu single atoms anchored in the nitrogen cavities of g-C_3_N_4_ are highly active and selective for the production of CH_4_. For the Cu_0.05_-CN catalyst with a coordination of N atoms in nitrogen cavities and a chemical state of dominant Cu^+^, the most optimal Cu-CN catalyst, presented the highest activity and CH_4_ FE (49.04%) and reached 7.97 mA cm^−2^ at -1.2 V_RHE_ with a high CH_4_/C_2_H_4_ ratio of 9, which, to the best knowledge of authors, is the best CO_2_-to-CH_4_ performance on g-C_3_N_4_-supported catalysts for CO_2_RR. Combining experimental evidence and density functional theory (DFT) calculations, it is revealed that one Cu single atom in one nitrogen cavity and coordinated with 4 N atoms of g-C_3_N_4_ exhibit a preferred CH_4_ catalytic pathway than the C_2_H_4_ pathway, while the preferred pathway is reversed after forming an adjacent O-coordinated Cu atom. The coordination environment regulated Cu_x_-CN catalysts for high-efficiency CH_4_ production provide a feasible way to fabricate single-atom Cu active sites for efficient and selective CO_2_RR catalysis and offer some insights into the design of future electrocatalysts.

## Experimental Section

### Materials

Dicyandiamide (DCDA), copper (II) acetylacetonate (Cu(acac)_2_), Iron (III) acetylacetonate (Fe(acac)_3_), and nickel (II) acetylacetonate (Ni(acac)_2_) were purchased from Sinopharm Chemical Reagent Co., LTD. Potassium bicarbonate (ACS, 99.7–100.5%) and deuterlum oxide (D_2_O, 99.9 atom% D) were purchased from Shanghai Aladdin Biochemical Technology Co., Ltd. Nafion solution (5 wt% in mixture of lower aliphatic alcohols and water) was purchased from Sigma-Aldrich. Nafion 117 membrane was purchased from Shanghai Hesen Electric Co., LTD. Standard liquid products: methanol (> 99.9%), sodium formate (99.99%), ethanol (> 99.8% for GC), and n-propanol (99.99%) were purchased from Shanghai Macklin Biochemical Co., Ltd. Deionized water (18.2 MΩ cm^−2^) was used in this work. The gas products, including H_2_, CO, CH_4_, and C_2_H_4_, were calibrated using standard mixed gases purchased from Dalian Special Gases Co., LTD.

### Preparation of Catalysts

To synthesis Cu_x_-CN catalysts, 1 g of DCDA and certain amount of Cu(acac)_2_ were well mixed in a mortar with the mass ratio of Cu(acac)_2_ to DCDA of x (x = 0.01, 0.05, 0.2, and 0.5 in this work). The well-mixed and grinded powder in a quartz boat was placed in the middle of a tubular furnace. Then the calcination process starts from room temperature to 550 ℃ at 5 ℃ min^−1^ and holds at 550 ℃ for 4 h in Ar atmosphere. After cooling to room temperature, the product was grinded into fine powder. CN sample was prepared by the same procedure except for the absence of Cu(acac)_2_. Ni_x_-CN and Fe_x_-CN materials were synthesized by the same method using Ni(acac)_2_ and Fe(acac)_3_ as the metal sources.

In order to leach out the Cu in Cu_0.05_-CN catalyst for comparison, the Cu_0.05_-CN catalyst was washed with 1.0 M nitric acid for 12 h at room temperature. After washing with deionized water to neutral pH, the sample that labeled as 0.05-CN was obtained, in which almost all of the Cu metal was removed by acid treatment (Table S1).

### Characterization of Materials

Transmission electron microscope (TEM) images were acquired using JEOL 2100F operated at 200 kV. High-angle annular dark-field scanning transmission electron microscopy (HAADF-STEM) and elemental mapping analysis were performed on Talos F200S operated at 200 kV. Powder X-ray diffraction (XRD) was obtained on Bruker D2 Phaser with a 2θ ranging from 10° to 90° using a Cu Ka X-ray. X-ray photoelectron spectroscopy (XPS) measurements were taken on Escalab 250Xi device. ^1^H nuclear magnetic resonance (NMR) spectra were collected on Bruker AVANCE III 600 MHz nuclear magnetic resonance spectrometer. Solid-state ^13^C magic angle spinning nuclear magnetic resonance (MAS NMR) spectra were acquired with a Bruker AVIII400 spectrometer with a 4 mm MAS BB-1H probe at frequency of 100.63 MHz. Elemental analysis (EA) was performed with Elmentar Vario EL III elemental analyzer to quantitatively determine the elemental content of C, H, and N. Fourier transform infrared (FTIR) spectrum was measured with a Nicolet 6700 FTIR spectrophotometer in the range of 400–4000 cm^−1^ using KBr pellet technique. Spherical aberration-corrected transmission electron microscope (AC-TEM) was performed on Themis ETEM (Thermo Fisher Scientific) transmission electron microscope. The measurements of X-ray absorption spectroscopy (XAS) at the Cu K-edge containing the X-ray absorption near-edge structure (XANES) and extended X-ray absorption fine structure (EXAFS) were taken at the beamline BL14W1 of Shanghai Synchrotron Radiation Facility (SSRF), China. The data processing of XAS measurements was performed using the Demeter software package. Nitrogen sorption–desorption measurements were taken at Micromeritics ASAP2046 machine. Before the measurements, the samples were degassed in a vacuum at 180 °C for at least 10 h. The Brunauer–Emmett–Teller (BET) method was utilized to calculate the specific surface areas using the adsorption data at P/P_0_ = 0.02—0.20. The pore size distribution was calculated from the adsorption branch by using the Barrett–Joyner–Halenda (BJH) model.

Electrochemical in situ attenuated total reflection Fourier transform infrared (ATR-FTIR) reflection spectroscopy was investigated on a Fourier transform infrared spectrometer (FTIR, Nicolet iS50, Thermo Fisher Scientific) equipped with a liquid nitrogen-cooled mercury cadmium tellurid (MCT) detector. The measurement was conducted in a homemade electrochemical cell equipped with a Pt-mesh and an Ag/AgCl as counter and reference electrodes, respectively. An Au-coated Si crystal loaded with catalysts was embed into the bottom of the cell to serve as the working electrode. CO_2_-saturated 0.1 M KHCO_3_ is used as the electrolyte for the in situ ATR-FTIR measurement, during which CO_2_ gas is continuously bubbled. Chronoamperometry is used for the in situ CO_2_RR test, and the spectrum is collected by 32 scans with 4 cm^−1^ resolution. All spectra were subtracted with the background.

### Electrode Preparation

Cu_x_-CN catalyst (1 mg) and carbon black (0.25 mg) were dispersed in methanol solution (190 uL). Subsequently, 10 uL of Nafion (5 wt%) was added, followed by ultrasonication for at least 1 h. Then 6 uL catalyst ink was dropped onto a L-type glass carbon electrode with a diameter of 4 mm (geometric area: ~ 0.1256 cm^2^) using a pipette and dried under ambient air. The catalyst loading was about 0.24 mg cm^−2^. The catalysts were electrochemically activated in CO_2_-saturated 0.1 M KHCO_3_ solution by CV (10 scans) from − 0.5 to -1.5 V_RHE_.

### Electrochemical Reduction of CO_2_

CO_2_ electrolysis was carried out in a gastight, custom-made two-compartment cell, in which the working electrode was separated from the counter electrode by a Nafion 117 membrane. Ag/AgCl (saturated KCl) was used as the reference electrode, and the three-electrode setup was connected to a potentiostat (Biologic VMP3). A 0.1 M KHCO_3_ electrolyte solution was used for all CO_2_RR tests, and the electrolyte in the cathodic compartment was stirred at a constant rate of ~ 300 rpm during electrolysis. Before CO_2_RR, the catholyte was bubbled with CO_2_ (99.99%) for 30 min to reach saturation, and CO_2_ was kept purging into the cathodic compartment at 10 sccm with stirring at ~ 500 rpm during the CO_2_RR. To determine the Faradaic efficiencies (FEs) of the reduced products, chronoamperometry was performed for 1 h at constant iR-corrected potentials. For the long-term stability test, the CO_2_-saturated 0.1 M KHCO_3_ electrolyte was extracted and replaced every 4 h to determine the liquid products. The FEs of gas products were calculated and recorded when the FEs were stable during the last 3 h.

All potentials were measured against an Ag/AgCl reference electrode (3.5 M KCl, stored in a 3.5 M KCl solution before and after use) and converted to the reversible hydrogen electrode (RHE) scale by

1 $$ E_{{{\text{RHE}}}} = E_{{\text{Ag/AgCl}}} + 0.2046 + 0.059 { \times }~ {\text{pH}} $$

The resistance between the reference and working electrodes was measured by potential electrochemical impedance spectroscopy (PEIS) and the ohmic drop was compensated automatically by the software (80%) and the rest 20% remained uncompensated. All the potentials in the text were iR-corrected if not otherwise specified. Generally, the value of resistance (R) was about 110–115 Ω in CO_2_-saturated 0.1 M KHCO_3_.

### Analysis of Gas and Liquid Products

Gas products from the cathodic compartment during CO_2_RR were analyzed using a GC-2014 (Shimadzu) equipped with a TCD detector and two FID detectors, one of which was coupled with a methanizer to detect CO concentration. High-purity Ar (99.999%) was used as the carrier gas. The calibration curves of gas and liquid products can be referred to our previous publication [3].

The Faradaic efficiencies of the gas products were calculated by the GC data using the following equation:

2 $$  FE_{g} \, = \,\frac{{Q_{g} }}{{Q_{{{\text{total}}}} }}\, \times \,100\%  = \frac{{\frac{\nu }{{60\;{\text{s}}/\min }}\, \times \,\frac{y}{{24.5\;{\text{L}}/{\text{mol}}}}\, \times \,n\, \times \,F}}{{\dot{J}_{{{\text{averge}}}} }}\, \times \,100\%  $$

where v is gas flow rate measured by a flowmeter (Beijing FLOWS instruments Co, Ltd), which is 10 sccm for all the tests,* y* the measured volumetric content of the gas product, n the number of electrons required to form the gas products, and n = 2, 2, 8, and 12 for H_2_, CO, CH_4_, and C_2_H_4_, respectively, F the Faraday constant (96,485 C mol^−1^), and *j* the average current density.

Liquid products were analyzed by a 600 MHz NMR spectrometer (Bruker Avance 3 HD 600 MHz) using a presaturation technique to suppress water peak. To take ^1^H NMR measurement, 800 uL of electrolyte sampled after CA or stability tests was mixed with 100 uL DMSO standard solution (100 ppm) and 100 uL D_2_O. The faradaic efficiencies of liquid products were calculated as follows:

3 $$ FE_{l} = \frac{{Q_{l} }}{{Q_{{{\text{total}}}} }}{ \times }100\% = \frac{{n_{l} { \times }n { \times }F}}{{Q_{{{\text{total}}}} }}{ \times }100\% $$

where n_l_ is the total content of certain liquid products in the catholyte, which was calculated by the concentration and the volume of the catholyte (45 mL), and n is the number of electrons required to form the liquid products, and n = 2, 6, 8, 12, and 18 for formate, methanol, acetate, ethanol, and 1-propanol, respectively.

### Computational Details

We carried out all the DFT calculations in the Vienna ab initio simulation (VASP5.4.4) code [37]. The exchange–correlation is simulated with PBE functional, and the ion–electron interactions were described by the PAW method [38, 39]. The vdWs interaction was included by using empirical DFT-D3 method [40]. The Monkhorst–Pack-grid-mesh-based Brillouin zone k-points are set as 3 × 3 × 1 for all periodic structure with the cutoff energy of 450 eV. The convergence criteria are set as 0.025 eV A^−1^ and 10^–4^ eV in force and energy, respectively. A 20 Å vacuum layer along the* z* direction is employed to avoid interlayer interference.

The free energy calculation of species adsorption (Δ*G*) is based on Nørskov et al.’s hydrogen electrode model [41].

4 $$ \Delta G = \Delta E + \Delta E_{{{\text{ZPE}}}} - T\Delta S $$

Herein Δ*E*, Δ*E*_ZPE_, and Δ*S*, respectively, represent the changes of electronic energy, zero-point energy, and entropy that caused by adsorption of intermediate. The entropy of H^+^ + e^−^ pair is approximately regarded as half of H_2_ entropy in standard condition [42].

## Results and Discussion

### Synthesis and Characterizations of Cu_x_-CN Catalysts

**Fig. 1:**
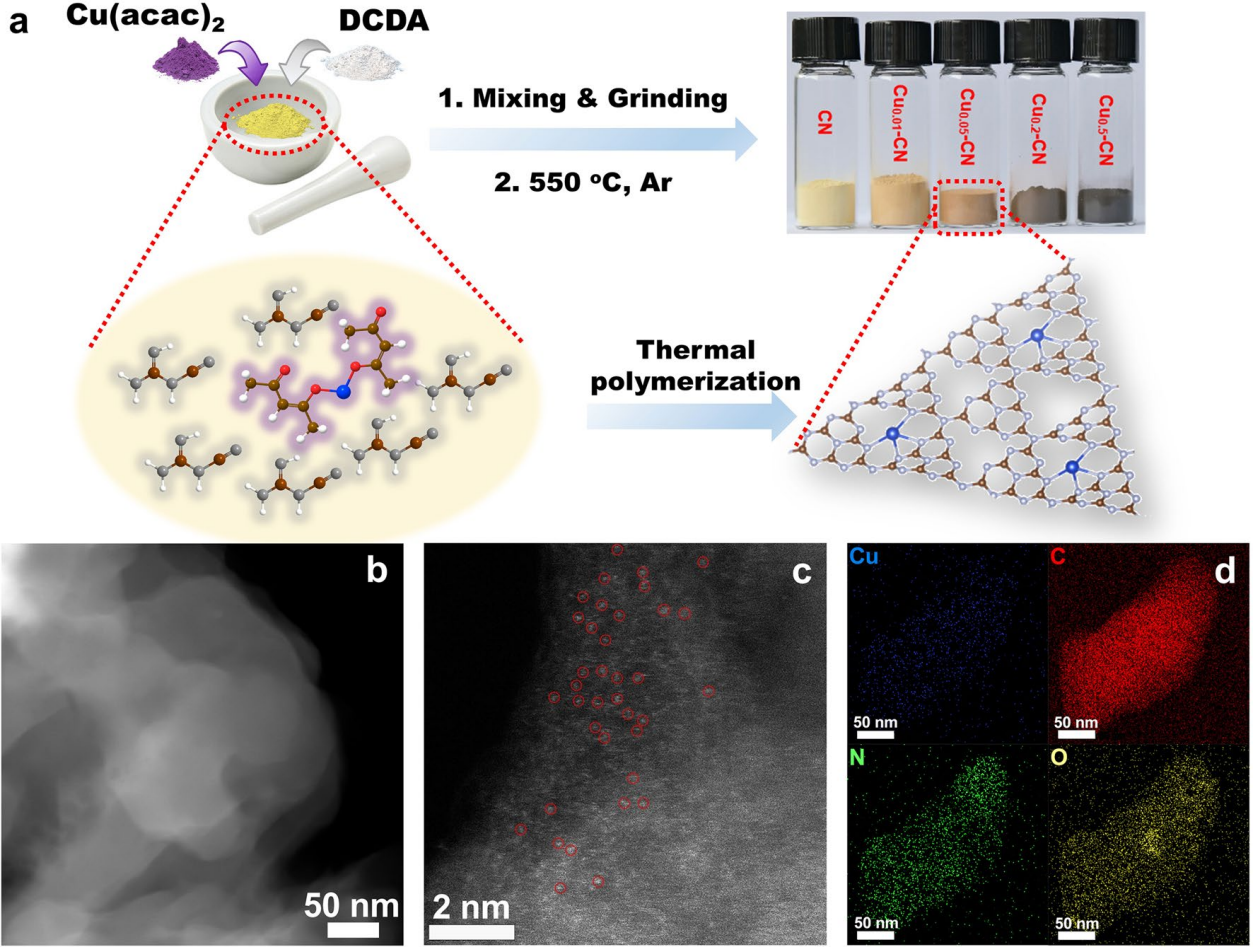
**a** Schematic illustration of the synthesis of Cu_x_-CN catalysts and the optical photographs of the as-synthesized Cu_x_-CN catalysts. **b** HAADF-STEM image, **c** aberration-corrected HAADF-STEM image, and **d** EDS mapping of Cu_0.05_-CN catalysts

The single Cu atoms anchoring in the nitrogen cavities of g-C_3_N_4_ catalysts with tailoring Cu coverage and coordination (Cu_x_-CN) were synthesized via an in situ thermal polymerization method by using dicyandiamide (DCDA) and copper (II) acetylacetonate (Cu(acac)_2_) as precursors for g-C_3_N_4_ host and Cu active species, respectively (Fig. 1a). In the synthesis, Cu(acac)_2_ and DCDA with a mass ratio of x (x = 0.01, 0.05, 0.2, and 0.5) in Cu_x_-CN catalysts were mixed and then calcined under N_2_ at 550 ℃ to obtain the Cu_x_-CN catalysts. The pure g-C_3_N_4_ (denoted as CN) is yellowish powders. With the increase in Cu contents in Cu_x_-CN catalysts, the color gradually turns into dark brown (Fig. 1a).

The morphology of the catalysts and the dispersion state of Cu species are first characterized. The TEM and high-angle annular dark-field scanning transmission electron microscopy (HAADF-STEM) images of both the CN and the Cu_x_-CN catalysts (Figs. 1b and S1-S2) confirm their stacking layered structures and exclude the existence of crystalline or aggregated Cu species in these catalysts. The stacked layers gradually became fragmentary with the increasing Cu content in Cu_x_-CN catalysts, demonstrating the increasing surface area and pore structure. Thus, the as-prepared material is investigated by the N_2_ adsorption–desorption measurements. As expected, after loading Cu the surface area and porosity of Cu_x_-CN catalysts increase significantly as compared with the pristine CN sample (Fig. S3). As observed on the Cu_0.05_-CN catalyst by aberration-corrected HAADF-STEM, the bright dots scatteringly distribute in the g-C_3_N_4_ host, indicating the presence of isolated Cu atoms in the catalyst (Fig. 1c). Interestingly, even at high Cu contents, isolated Cu atoms appeared at a high density rather than forming crystalline species or aggregated clusters, for example, in the Cu_0.5_-CN catalysts (Fig. S2c), which is consistent with the TEM results. The uniform distribution of Cu, C, N, and O elements in the Cu_0.05_-CN catalyst is further verified by the elemental mappings (Figs. 1d and S4). The high density of the atomic sites shortens the distance between single atoms, which may impact on both the activity and the selectivity of the catalysts [25, 43]. Besides, this facile strategy can be extended to other transition metals to form g-C_3_N_4_-supported single metal atoms, such as Ni and Fe (Figs. S5-S6).

**Fig. 2:**
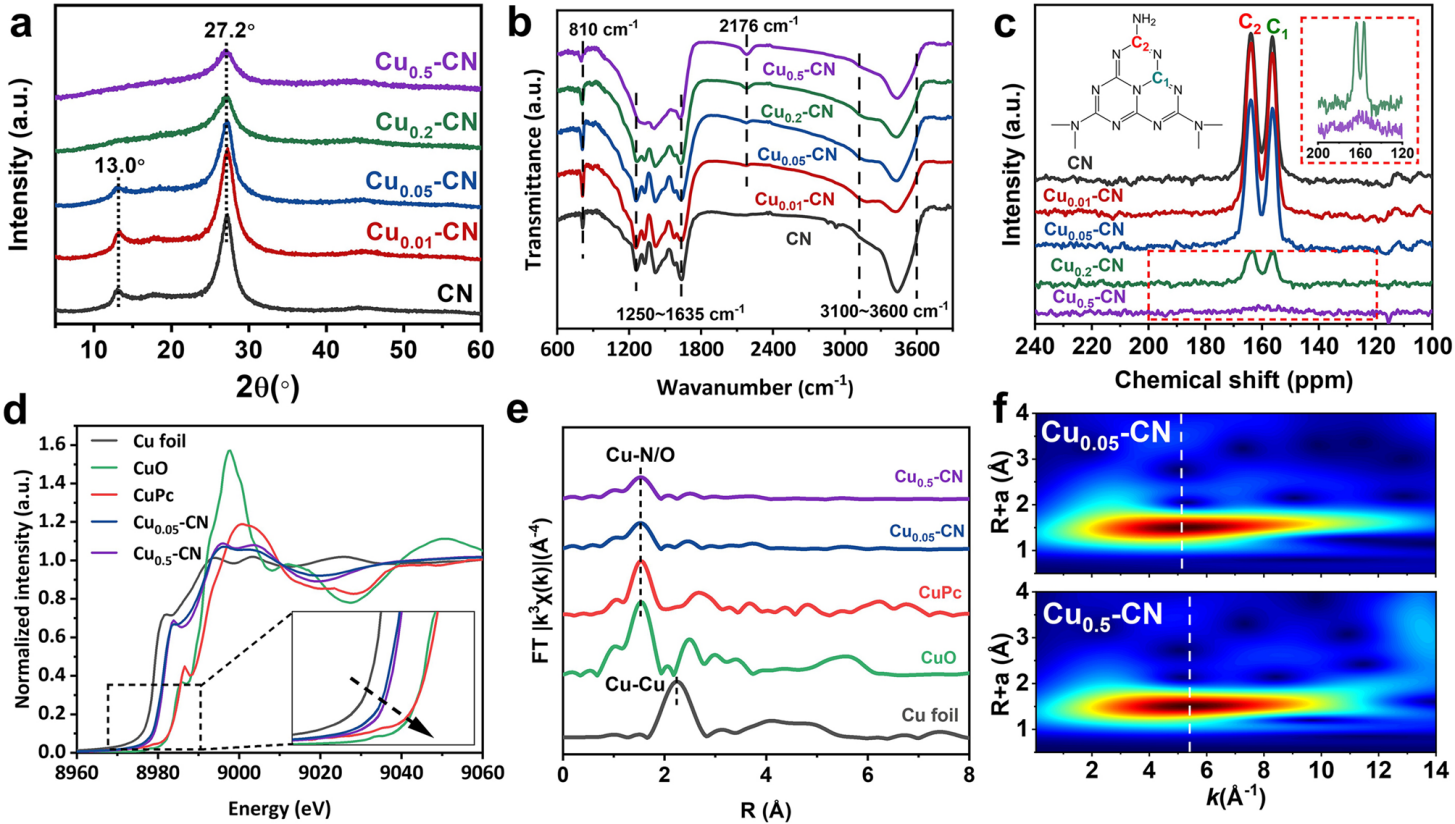
**a** XRD patterns of Cu_x_-CN catalysts. **b** FTIR spectra of Cu_x_-CN catalysts. **c** Solid-state ^13^C NMR spectra of Cu_x_-CN catalysts. **d** Cu K-edge XANES spectra of Cu foil, Cu_0.05_-CN, Cu_0.5_-CN, CuPc, and CuO samples. **e** Fourier transform (FT) EXAFS spectra of Cu K-edge of Cu foil, Cu_0.05_-CN, Cu_0.5_-CN, CuPc, and CuO samples. **f** Wavelet transform (WT) of Cu_0.05_-CN and Cu_0.5_-CN catalysts

The XRD patterns of the CN, the Cu_0.01_-CN, and the Cu_0.05_-CN catalysts exhibit the (100) and (002) peaks of g-C_3_N_4_ appeared at 13.0° and 27.2°, corresponding to the repeating in-plane melem units and the stacking of graphite-like layer structure, respectively (Fig. 2a) [28]. For the Cu_0.2_-CN and Cu_0.5_-CN catalysts, the intensity of (002) peak decreased and the (100) peak disappeared, indicating the loss of periodical repeatability of the in-plane melem units in these two catalysts. No phase of Cu species appeared in all catalysts, which excludes the existence of crystalline Cu species and indicates the likely chemically coordination of Cu into the g-C_3_N_4_ host in the form of Cu-N_y_ structure (y is the coordinated N number) [28]. The FTIR spectra of all samples exhibit a broad peak between 3100 and 3600 cm^−1^ that can be attributed to N–H and O–H stretching [44, 45], while the weak peaks appear at ~ 2176 cm^−1^ can be assigned to the stretching of triple C≡N bond, which is considered as defects within g-C_3_N_4_ [46]. The increasing intensity of C≡N peaks support the fact that the defect increases with increasing Cu content in Cu_x_-CN catalysts. The typical bands of the stretching vibrations of C-N heterocycles and breathing mode of s-triazine units appears in at ~ 1250–1635 and at ~ 810 cm^−1^, respectively [47–49]. Notably, at low Cu loads, these two characteristic FTIR spectra of the Cu_0.01_-CN and Cu_0.05_-CN catalysts are almost identical with that of the pure g-C_3_N_4_ (Fig. 2b), indicating the well-preserved g-C_3_N_4_ frameworks. On the contrary, these characteristic bands are significantly weakened in the Cu_0.2_-CN and Cu_0.5_-CN catalysts, indicating the high Cu loads in the catalysts can partially damage of the C-N heterocyclic structure [50]. To further confirm the local structures of the Cu_x_-CN catalysts, solid-state ^13^C nuclear magnetic resonance (^13^C NMR) spectra were carried out on the Cu_x_-CN with various Cu contents (Fig. 2c). Two distinct peaks at chemical shift of 156.3 and 164.0 ppm, which are assigned to the C atoms connected to different N atoms in C-N heterocycle [49], are observed in the CN, Cu_0.01_-CN, and Cu_0.05_-CN samples, indicating the skeleton structure of g-C_3_N_4_ are well retained and the introduced Cu atoms are likely to incorporate into the nitrogen cavities among C-N heterocyclic structures in these samples. However, the two peaks dramatically attenuate for the Cu_0.2_-CN and Cu_0.5_-CN catalysts, demonstrating the destruction of g-C_3_N_4_ framework by increasing Cu(acac)_2_ content in the preparation. Obviously, the XRD, FTIR, and ^13^C NMR results explicitly suggest that the basic structure units and framework of the g-C_3_N_4_ host are well retained for Cu_0.01_-CN and Cu_0.05_-CN catalysts with low Cu loads, which is likely associated with the preferential incorporation of isolated Cu atoms in the nitrogen cavities of g-C_3_N_4_ with Cu–N structures. On the other hand, the destructed C-N heterocycles and g-C_3_N_4_ framework of Cu_0.2_-CN and Cu_0.5_-CN catalysts indicate the changed atomic coordination at relatively high Cu loads.

To get a clearer understanding on the chemical states and coordination of Cu atoms in the Cu_x_-CN catalysts, XAFS measurements were taken together with Cu foil, copper (II) phthalocyanine (CuPc), and CuO as references. As illustrated by the X-ray absorption near-edge structure (XANES) in Fig. 2d, the positions of Cu K-edge for the Cu_0.05_-CN and Cu_0.5_-CN catalysts are close, which are located between the Cu K-edge edges of Cu foil and CuO. The average oxidation states of Cu in the Cu_0.05_-CN and Cu_0.5_-CN catalysts are found to be close to + 1 by fitting (Fig. S7) [51]. The Fourier transformed extended EXAFS spectra with *k*^3^-weight of Cu_0.05_-CN, Cu_0.5_-CN, and the references are depicted in Fig. 2e. It is found that the Cu_0.05_-CN and Cu_0.5_-CN catalysts exhibited only one dominating peak around 1.53 Å, which could be assigned to the scattering of either Cu–N or Cu–O coordination [24]. As references, CuO shows a main peak attributed to Cu–O at 1.53 Å while the Cu-Cu coordination in Cu foil is at 2.24 Å. These results further verify that no Cu-Cu interaction existed but only the isolated Cu atoms in the Cu_x_-CN catalysts, even for Cu_0.5_-CN with high Cu loads. Notably, the Cu_0.5_-CN catalyst exhibited a lower intensity at 1.53 Å than that of the Cu_0.05_-CN catalyst, suggesting a possible different coordination environment achieved at higher Cu contents [52, 53]. Due to the great resolution in both k and R spaces, the wavelet transform of Cu K-edge EXAFS spectra were performed to investigate the atomic configuration of Cu_0.05_-CN and Cu_0.5_-CN catalysts (Fig. 2f). summits at 5.2 and 5.4 Å^−1^ were identified for Cu_0.05_-CN and Cu_0.5_-CN, respectively, in the k space. Although the wavelet transform of Cu K-edge EXAFS is unable to discriminate the N and O coordination completely [24], these results confirm the coordination environment of the Cu single atoms in Cu_x_-CN can be varied as the variation of Cu loading amounts.

**Fig. 3:**
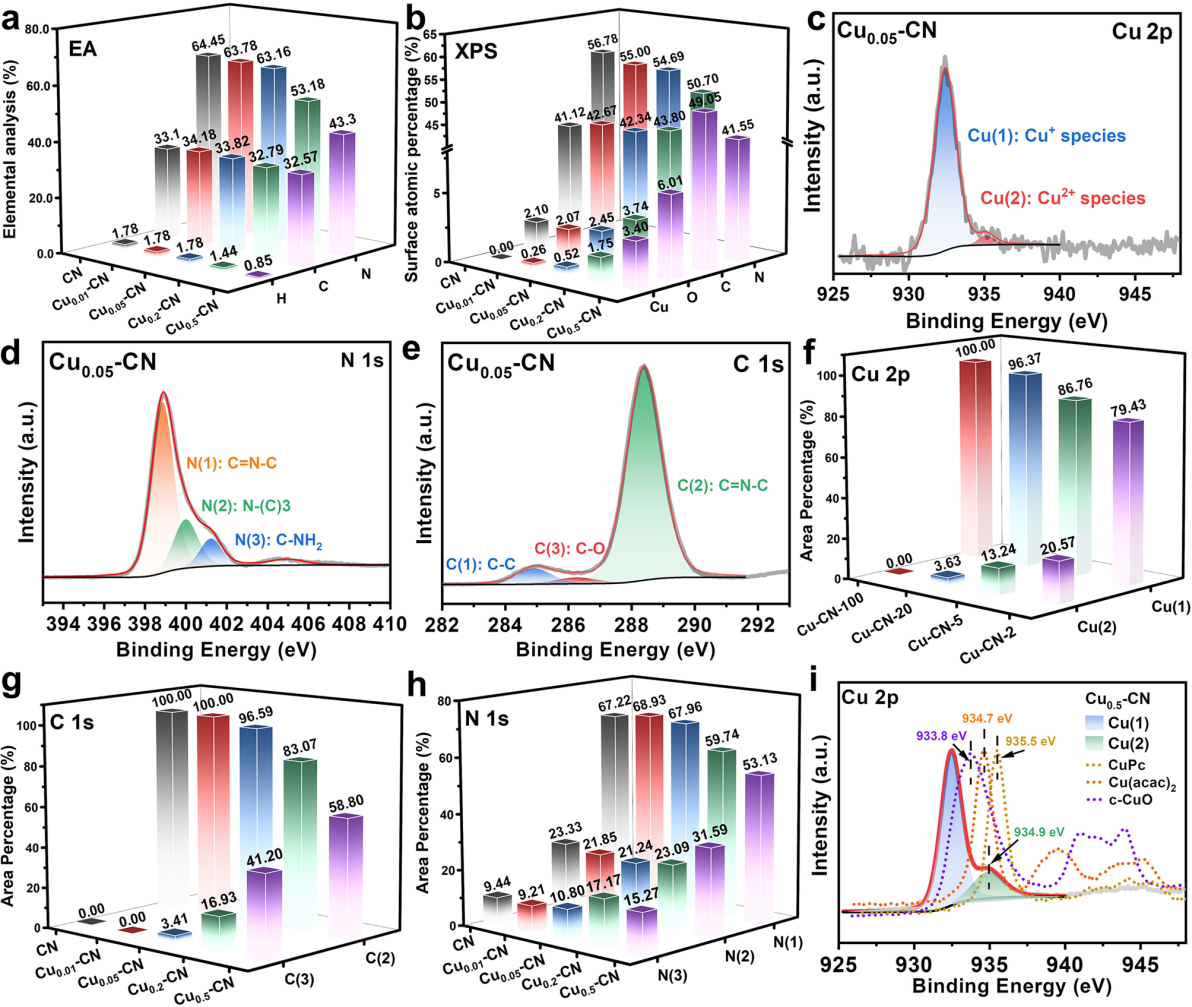
**a** Elemental analysis of the catalysts. **b** Surface composition of the catalysts determined by XPS. **c-e** Peak fitting of Cu 2*p*, N 1*s*, and C 1*s* spectra of the Cu_0.05_-CN catalyst. **f–h** Peak fitting summary of Cu 2*p*, C 1*s*, and N 1*s* spectra of the catalysts. **i** Comparison of Cu 2*p* spectra of the Cu_0.5_-CN catalyst with other cupric compounds

The composition, valence state, and detailed structural and binding information of the Cu_x_-CN catalysts were further investigated by various characterizations. To verify the actual Cu contents in each catalyst, inductively coupled plasma–atomic emission spectrometry (ICP-AES) was employed. It reveals that the actual Cu contents are proportional to the Cu(acac)_2_ addition amounts, and the Cu_0.05_-CN catalyst had a Cu content of 0.954 wt% (Table S1). The compositions of the CN and Cu_x_-CN catalysts were then probed by an elemental analysis (EA). As shown in Fig. 3a, the CN and Cu_x_-CN catalysts possess similar C content, while the N and H contents decrease distinctly for the Cu_0.2_-CN and Cu_0.5_-CN catalysts. Similarly, the significant decrease of N content in these two catalysts was also observed by the atomic percentage determined by XPS (Figs. 3b and S8), accompanying with the increases in Cu and O contents. The composition variations indicate that the Cu atoms are successfully introduced in g-C_3_N_4_ host accompanied by the introduction of O atoms, which is hypothesized to be caused by the use of oxygen-containing copper salt (copper acetylacetonate) in the polymerization process of g-C_3_N_4_.

The bonding and chemical states of the elements in CN and Cu_x_-CN catalysts are analyzed by the XPS spectra collected on the Cu, C, and N elements of all catalysts. The Cu 2*p* peaks can be fitted with two peaks (Figs. 3c and S9). The Cu(1) peak at about 932.4 eV is predominant in the Cu_0.01_-CN and Cu_0.05_-CN catalysts and can be ascribed to Cu^0^ or Cu^1+^ species, while the Cu(2) peak at about 935.0 eV ascribed to Cu^2+^ species increases significantly in the Cu_0.2_-CN and Cu_0.5_-CN catalysts with higher Cu contents (Fig. S6d-e). Further, according to the XANES results and the Cu LMM Auger spectra of Cu_x_-CN catalysts, it can be concluded that Cu(1) peaks in Cu_x_-CN should be ascribed to Cu^1+^ species, as the overwhelming Cu^1+^ peaks and negligible Cu^0^ peaks in the Cu LMM spectra (Fig. S9f) [54]. The N 1*s* spectra can be deconvoluted into three states of N(1), N(2), and N(3) at about 398.7, 399.8, and 401.1 eV, respectively (Figs. 3d and S10), as well as a small peak at 404.9 eV, which corresponds to the π-excitation. The N(1), N(2), and N(3) peaks can be attributed to the *sp*^2^ hybridized nitrogen in C-N heterocycles (C = N–C), the tertiary N (N-(C)3), and the N in amino functional groups (C-NH_2_), respectively [55]. The C 1*s* peaks can also be fitted with three peaks denoted as C(1), C(2), and C(3) at about 284.8, 288.3, and 286.6 eV, respectively (Figs. 3e and S11). The C(1) peak can be ascribed to graphitic carbon and used for calibration. The C(2) peak is the main component of C 1*s* spectra for all catalysts, which should be the C atoms in the C-N heterocycles (C = N–C). The C(3) state gradually emerges and rises with the increase of Cu contents in Cu_x_-CN catalysts, which may attribute to the C in a C-O structure [55–57].

The fitting results of the Cu 2*p*, C 1*s*, and N 1*s* spectra are summarized in Fig. 3f-h. In accordance with the structural characterization of the catalysts in Fig. 2, the fitting results show that the species and ratio of C and N are almost unaffected for the Cu_0.01_-CN and Cu_0.05_-CN catalysts. Combined the results of XAFS measurements, it is reasonable that the isolated Cu atoms preferentially embed nitrogen cavities to form Cu–N coordination structures with little effects on the C-N heterocyclic structure in these two catalysts. However, with the increasing addition of Cu(acac)_2_ in the preparation, the significantly reduced N(1) and C(2) peaks further confirm the destruction of the C-N heterocyclic structure of the g-C_3_N_4_ host in the Cu_0.2_-CN and Cu_0.5_-CN catalysts. Meanwhile, the concurrent increases of the Cu(2) species and the O atomic ratio for the Cu_0.2_-CN and Cu_0.5_-CN catalysts imply the emerging Cu^2+^ species is related to the increasing C-O structures, which is evidenced by increased C(3) peaks and the O 1*s* spectra (Figs. 3g and S12). Further, by comparing the Cu 2*p* spectra of the Cu_0.5_-CN catalyst with some references, including commercial CuO powder, Cu(acac)_2_, and CuPc, it is found that the binding energy of Cu^2+^ species in the Cu_0.5_-CN catalyst is close to Cu(acac)_2_ (Fig. 3i), indicating the structure of the emerging Cu^2+^ sites in the Cu_0.2_-CN and Cu_0.5_-CN catalysts are likely the O-coordinated structure [58]. Therefore, the possible evolution of the atomic configurations of the Cu_x_-CN catalysts can be drawn from the above analysis. In the Cu_0.01_-CN and Cu_0.05_-CN catalysts, the atomic Cu sites predominantly embed in the nitrogen cavities of the g-C_3_N_4_ host, forming Cu–N coordination without affecting its framework structure. However, in the Cu_0.2_-CN and Cu_0.5_-CN catalysts, the O-coordinated Cu species emerge substantially accompanied with the partial destruction of the C-N heterocycles and g-C_3_N_4_ framework.

### Catalytic Performance of CO_2_RR

**Fig. 4:**
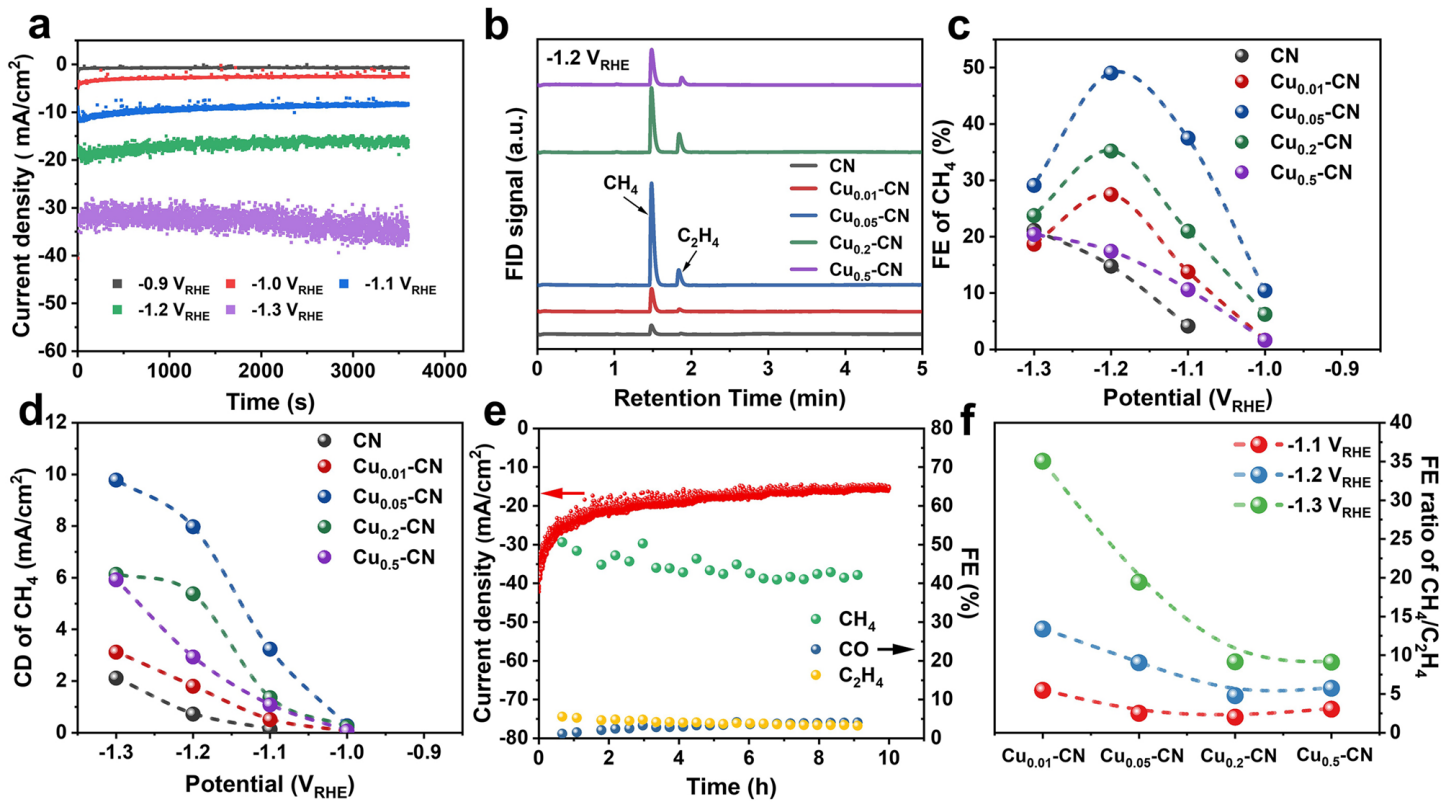
CO_2_RR performance of CN and Cu_x_-CN catalysts. **a** Typical chronoamperometry curves at different potentials on Cu_0.05_-CN catalysts. **b** FID signals of GC showing the main gaseous CO_2_RR products of CH_4_ and C_2_H_4_ on Cu_x_-CN catalysts. **c** FE of CH_4_ at different potentials on Cu_x_-CN catalysts. **d** Current density (CD) of CH_4_ at different potentials on Cu_x_-CN catalysts. **e** Catalytic stability of Cu_0.05_-CN catalyst during 10 h of CO_2_RR test at -1.2 V_RHE_. **f** FE ratio of CH_4_/C_2_H_4_ on Cu_x_-CN catalysts at different potentials

The CO_2_RR performance of the CN and Cu_x_-CN catalysts was evaluated in a H-cell with 0.1 M KHCO_3_ as the electrolyte. The potential dependent FE of different products on the CN and Cu_x_-CN catalysts was collected by a chronoamperometry test of 1 h in the potential range from -0.9 to -1.3 V_RHE_ (Fig. 4a). The gaseous products account for the major reduction products on all catalysts (Figs. 4b and S13-S14). Compared with the pristine g-C_3_N_4_, the Cu_x_-CN catalysts exhibited higher activity and selectivity toward CH_4_ in the tested potential range. The highest FE of 49.04% at -1.2 V_RHE_ for CH_4_ reached on the Cu_0.05_-CN catalyst with a CH_4_ current density of 7.97 mA cm^−2^, which further increased to 9.78 mA cm^−2^ at -1.3 V_RHE_ (Fig. 4c-d). Although the CN catalyst also exhibits certain activity toward CO_2_RR in the potential range, it only shows quite low CH_4_ activity and selectivity with a maximum CH_4_ current density of 2.11 mA cm^−2^ at -1.3 V_RHE_. Clearly, the enhanced CH_4_ production obtained on the Cu_x_-C_3_N_4_ catalysts indicates the incorporated Cu sites in these catalysts are active sites for CO_2_RR catalysis. Probably contributed by the stable configuration of single Cu atoms in the Cu_0.05_-CN catalysts (Fig. S15), the catalytic stability up to 10 h of continuous CO_2_RR test was performed on the Cu_0.05_-CN catalyst at -1.2 V_RHE_, which showed a slight decrease within the stability test (Fig. 4e).

To gain further insight into the improved CH_4_ activity and selectivity and elucidate the role the atomic Cu sites in the Cu_0.05_-CN catalyst, temperature-programmed CO desorption and control experiments were carried out. The larger desorption peak area and stronger chemisorption of CO on the Cu_0.05_-CN catalyst than CN indicate the increased CO binding sites and enhanced CO binding strength resulted by Cu incorporation (Fig. S16a) [36]. To probe the role of the single Cu atoms for CO_2_RR-to-CH_4_, the Cu_0.05_-CN catalyst was washed by 1.0 M nitric acid for 12 h at room temperature to obtain 0.05-CN sample and the almost all of the Cu metal was removed by acid treatment (Table S1). Compared with the Cu_0.05_-CN catalyst, the 0.05-CN catalyst exhibits much lower current density without CH_4_ product at -1.2 V_RHE_ (Fig. S16b-c). In addition, the effect of carbon black in the preparation of catalysts ink on the CO_2_RR is also excluded (Fig. S16d). Therefore, it is believed that the high CO_2_RR-to-CH_4_ activity and selectivity on the Cu_0.05_-CN catalyst are originated from the single Cu sites embedded into the nitrogen cavities in the g-C_3_N_4_ host. However, it should be noted that the semiconductor characteristic of g-C_3_N_4_ host lead to the poor charge transfer ability of the Cu_x_-CN catalysts, although the charge transfer resistance of CN can be reduced slightly by Cu doping (Fig. S17) [30]. Therefore, improving the conductivity may be a promising for further boosting the CO_2_RR performance of Cu_x_-CN catalysts.

### Discussions

It has been reported that the isolated Cu sites tended to convert CO_2_ into C_1_ products in CO_2_RR, such as CO, CH_4_, and CH_3_OH [16, 19, 25, 59]. On the other hand, the C_2+_ products could also be obtained on Cu single-atom catalysts if the atomically dispersed Cu reversibly transformed into clusters or nanoparticles under reaction conditions or additional active centers acted collaboratively with single Cu sites [21, 22]. Furthermore, the density of Cu sites and their distance played a key role in the selectivity between the CH_4_ and the C_2_H_4_ products and the ratio of CH_4_/C_2_H_4_ as the adjacent Cu-N_2_ sites enabled the C–C coupling, which is prerequisite for C_2_H_4_ formation [25]. Given two types of controllable and adjustable Cu sites in the Cu_x_-CN catalysts in this work, the FE ratios of CH_4_/C_2_H_4_ on Cu_x_-CN catalysts are compared at different potentials. As shown in Fig. 4f, the FE ratio of CH_4_/C_2_H_4_ increased with the decrease of potentials for all catalysts. The Cu_0.01_-CN catalyst with the lowest Cu content and exclusive single-atom Cu sites shows the highest FE ratio of CH_4_/C_2_H_4_ of 35.03 at -1.3 V_RHE_. Such high FE ratio of CH_4_/C_2_H_4_ outperforms most reported results (Fig. S18), which is suggested to ascribed to the highly selective toward CH_4_ of the single Cu atomic sites in nitrogen cavities and the long distance between these sites of the Cu_0.01_-CN catalyst with low Cu load. The Cu_0.05_-CN catalyst shows a CH_4_/C_2_H_4_ FE ratio of 9.03 at -1.2 V_RHE_, at which the highest FE of CH_4_ is achieved. The decreased CH_4_/C_2_H_4_ FE ratio on the Cu_0.05_-CN catalyst is hypothesized to be resulted from the shortened distance between single Cu atoms at increasing Cu loads and the small amount of Cu^2+^ species in the Cu_0.05_-CN catalyst [25]. Therefore, the high CH_4_/C_2_H_4_ FE ratio on the Cu_0.01_-CN catalyst and the outstanding CO_2_RR-to-CH_4_ performance on the Cu_0.05_-CN catalyst support the hypothesis that the single Cu atoms embedded into the nitrogen cavities of the g-C_3_N_4_ host are active sites for CH_4_ formation.

**Fig. 5:**
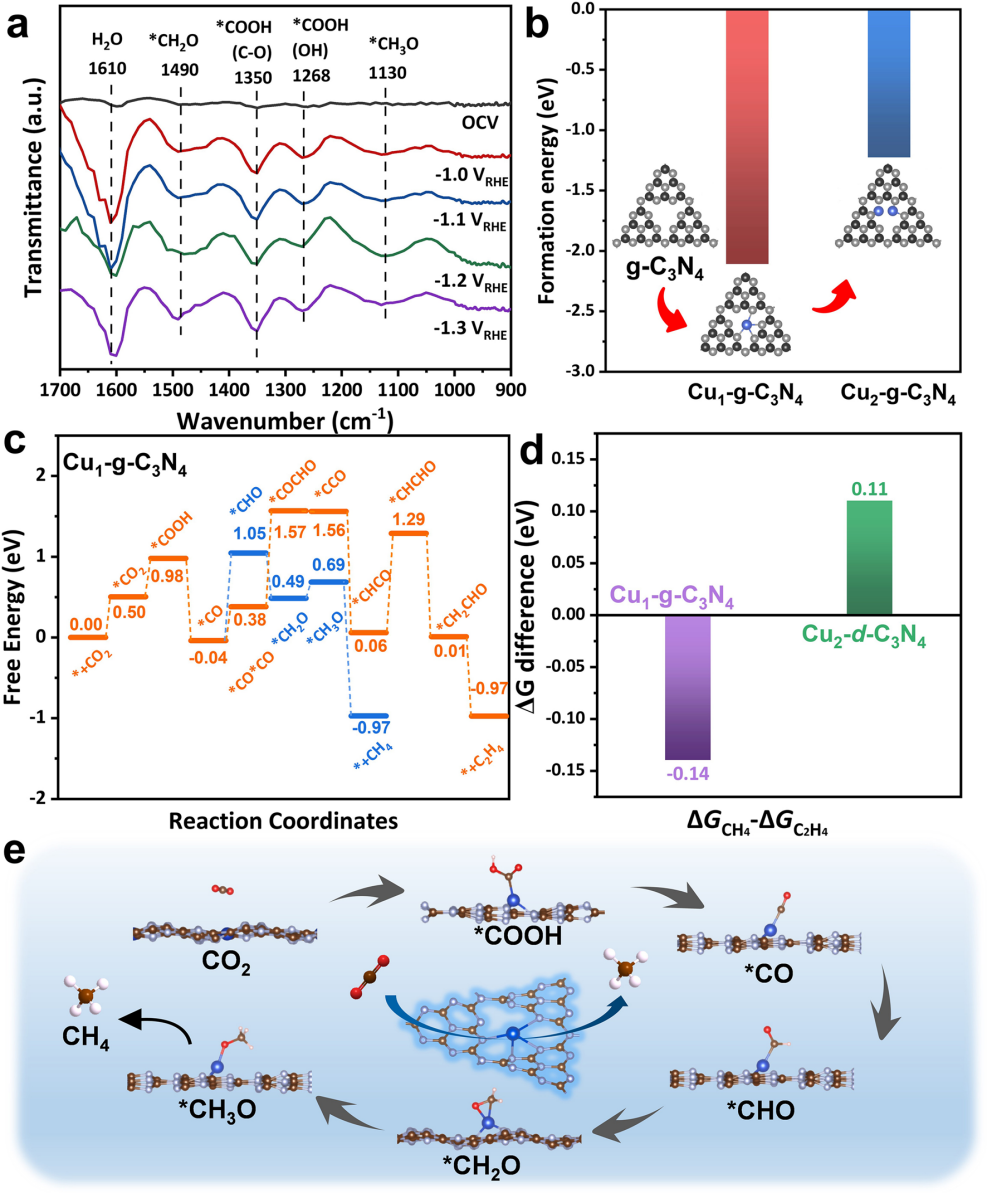
Mechanism and theoretical study. **a** In situ ATR-FTIR spectra of Cu_0.05_-CN catalysts at decreasing potentials in a CO_2_-saturated 0.1 M KHCO_3_ electrolyte. **b** Calculated formation energies of single and dual Cu sites in the nitrogen cavity of g-C_3_N_4_. **c** Free energy diagram of CO_2_ to CH_4_ and C_2_H_4_ on Cu_1_-g-C_3_N_4_. **d** Difference of free energy barriers between CH_4_ and C_2_H_4_ pathways on Cu_1_-g-C_3_N_4_ and Cu_2_-d-C_3_N_4_. **e** Proposed reaction pathway of CH_4_ formation on Cu_1_-g-C_3_N_4_

To explore the reaction mechanism of the CO_2_RR on Cu_x_-CN catalysts, in situ ATR-FTIR measurements were taken to identify the key intermediates during CO_2_RR in a homemade cell (Fig. S19). As shown in Fig. 5a, the peaks at 1268 and 1350 cm^−1^ can be ascribed to the *COOH intermediate, which is generally considered as the key intermediate for CO_2_ electrochemical conversion to CO and further reduction [60]. Notably, the signals at 1130 and 1490 cm^−1^, which can be assigned to *CH_2_O and *CH_3_O, respectively [61], intensify as the potentials decreased from -1.0 V_RHE_ to -1.2 V_RHE_. Therefore, the formation of CH_4_ on Cu_0.2_-CN catalysts is likely through the proton–electron transfer of *COOH, *CO, *CHO, *CH_2_O, and *CH_3_O in succession [60, 62].

To gain a comprehensive insight into the formation of atomic Cu sites and the structure–activity relationship of Cu_x_-CN catalysts, DFT calculations were then performed. Previously, both experimental and theoretical studies have demonstrated that the nitrogen cavity consists of six nitrogen atoms in g-C_3_N_4_, which is the prevailing and stable structure for accommodating single metal atoms [36, 63]. The calculation also shows that the formation energy of a Cu atom incorporated in one nitrogen cavity of g-C_3_N_4_ (Cu_1_-g-C_3_N_4_) is -2.11 eV (Fig. 5b), which is far less than that for forming two Cu atoms into one cavity (Cu_2_-g-C_3_N_4_). Furthermore, the experimental EXAFS spectrum can be fitted with great accuracy using Cu_1_-g-C_3_N_4_ model, giving the average bond length R of 1.92 Å and the coordination number N of about 3.3 for Cu–N bond (Fig. S20 and Table S2). The optimized structure shows that the incorporated Cu atom coordinates with four N atoms in the nitrogen cavities and the charge transfer occurs from Cu to N atoms (Fig. S21). The calculated free energy profile demonstrates that the rate determining step (RDS) for the conversion of CO_2_-to-CH_4_ is *CO → *CHO with a ΔG of 1.09 eV on the Cu_1_-g-C_3_N_4_ single active site, which is about 0.14 eV lower than the ΔG of RDS of the C_2_H_4_ pathway (Fig. 5c). Therefore, it is more energy favorable to form CH_4_ rather than other hydrocarbons on single Cu atomic sites hosted by g-C_3_N_4_. Based on the experimental observation, which shows the destructed g-C_3_N_4_ structure and the increasing O-coordinated Cu atoms in the Cu_x_-CN catalysts with increasing Cu loads, the model of Cu_2_-d-C_3_N_4_ consisted of adjacent Cu sites and damaged C-N heterocycles is built (Fig. S22). The free energy barrier of the RDS for the CH_4_ pathway is 0.11 eV higher than that for the C_2_H_4_ pathway on the dual-site Cu_2_-d-C_3_N_4_ (Fig. S23). According to the free energy differences between CH_4_ and C_2_H_4_ pathways (Fig. 5d), it is concluded that the CH_4_ pathway prevails over the C_2_H_4_ pathway on single Cu atomic sites in nitrogen cavities of g-C_3_N_4_, while the trend reverses on dual Cu sites on destructed g-C_3_N_4_ host. Therefore, the emergence of adjacent O-coordinated Cu sites in the Cu_x_-CN catalysts with high Cu loads suppresses the CH_4_ formation but facilitates the production of C_2_H_4_, as observed in experiments. Based on the mechanism and theoretical study, the CO_2_-to-CH_4_ pathway on the favorable Cu sites is proposed to proceed via the intermediates of *COOH, *CO, *CHO, *CH_2_O, and *CH_3_O (Fig. 5e).

## Conclusions

In this work, structurally stable single Cu atoms embedded in the nitrogen cavities of g-C_3_N_4_ frameworks have been successfully synthesized with controlled configurations and chemical states to catalyze CO_2_RR for CH_4_ production. High CH_4_ activity and selectivity and high FE ratio of CH_4_/C_2_H_4_ were achieved on the Cu_0.01_/Cu_0.05_-CN catalysts with predominant single Cu atomic sites. The increase of Cu contents in the Cu_x_-CN catalysts leads to the emerging of a second O-coordinated Cu sites accompanied with the structural destruction of g-C_3_N_4_ support, resulting in reduced CH_4_ activity and selectivity and thus decreased CH_4_/C_2_H_4_ ratio. This work provides a new strategy for constructing efficient single Cu atom catalysts for the conversion of CO_2_-to-CH_4_ and delivers some insights into the understanding on the regulation of Cu-based catalysts for CO_2_RR to achieve desired selectivity and products.

### Supplementary Information

Supplementary file 1 (PDF 1,877 kb)
