# Supplementary material for: Atomic Cu Sites Engineering Enables Efficient CO2 Electroreduction to Methane with High CH4/C2H4 Ratio
Source: Nanomicro Lett. 2023 Oct 26;15:238. doi: 10.1007/s40820-023-01188-1 (PMC10603021; doi:10.1007/s40820-023-01188-1)
Supplement: Supplementary file 1 — (PDF 1,877 kb) [file 40820_2023_1188_MOESM1_ESM.pdf]

Supporting Information for

Atomic Cu Sites Engineering Enables Efficient CO_2 Electroreduction to Methane with High CH_4/C_2H_4 Ratio

Minhan Li[1, 2, #], Fangzhou Zhang[1, #], Min Kuang[1], Yuanyuan Ma[1, *], Ting Liao[3], Ziqi Sun[3], Wei Luo[1], Wan Jiang[1], and Jianping Yang[1, *]

[1] Institute of Functional Materials, State Key Laboratory for Modification of Chemical Fibers and Polymer Materials, College of Materials Science and Engineering, Donghua University, Shanghai 201620, P. R. China

[2] College of Materials Science and Engineering, Zhengzhou University, Zhengzhou 450001, P. R. China

[3] School of Mechanical, Medical and Process Engineering, School of Chemistry and Physics, Queensland University of Technology, 2 George Street, Brisbane, QLD 4001, Australia

[#]Minhan Li and Fangzhou Zhang contributed equally to this work.

*Corresponding authors. E-mail: yyma@dhu.edu.cn (Yuanyuan Ma); jianpingyang@dhu.edu.cn (Jianping Yang)

Supplementary Figures and Tables

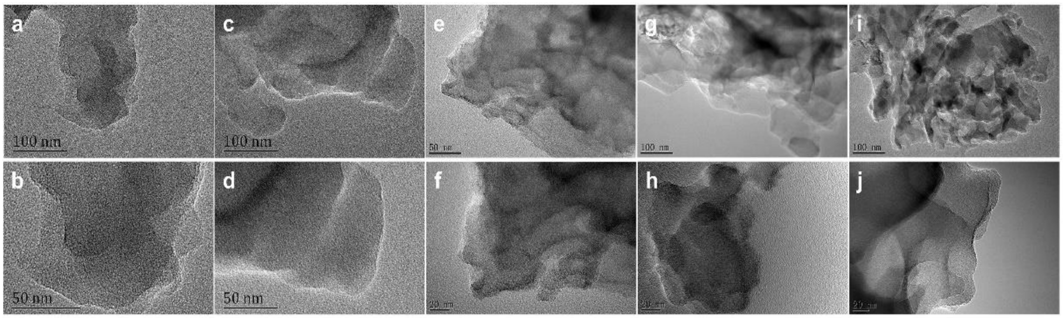

Fig. S1 TEM images of CN and Cu_x-CN catalysts. (**a-b**) CN. (**c-d**) $Cu_{0.01}$-CN. (**e-f**) $Cu_{0.05}$-CN. (**g-h**) $Cu_{0.2}$-CN. (**i-j**) $Cu_{0.5}$-CN

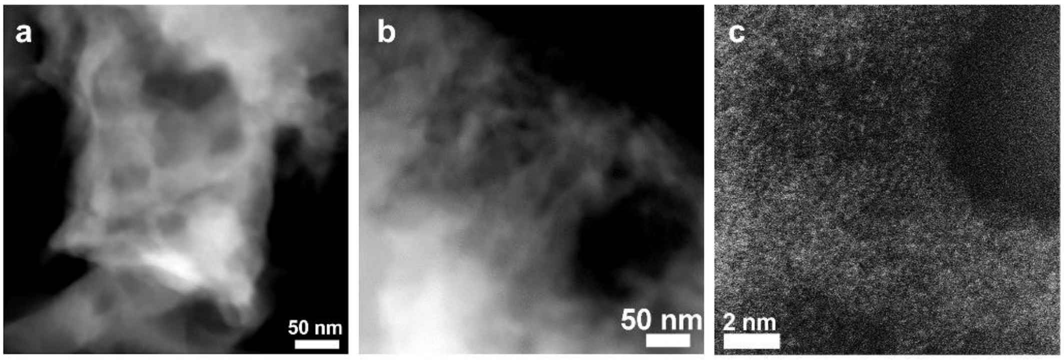

Fig. S2 The HAADF-STEM images of the $Cu_{0.2}$-CN (**a**) and $Cu_{0.5}$-CN catalysts (**b**). The aberration-corrected HAADF-STEM image of the $Cu_{0.5}$-CN catalyst

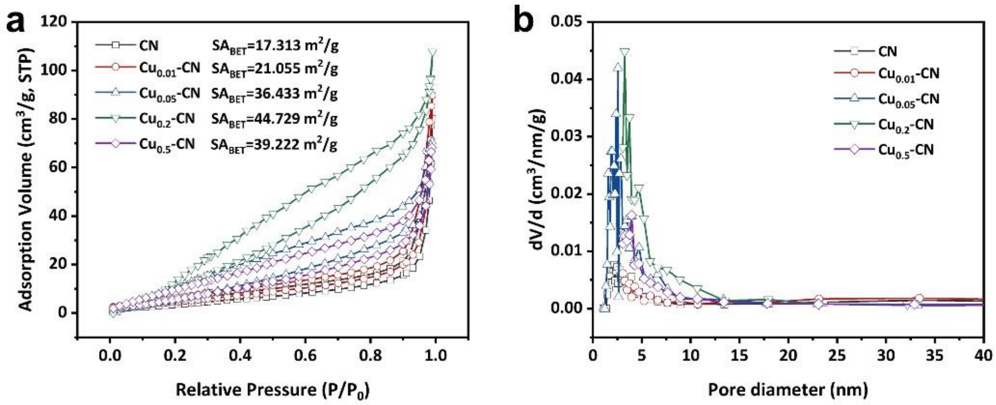

Fig. S3 Nitrogen sorption-desorption isotherms (**a**) and the corresponding pore size distribution (**b**)

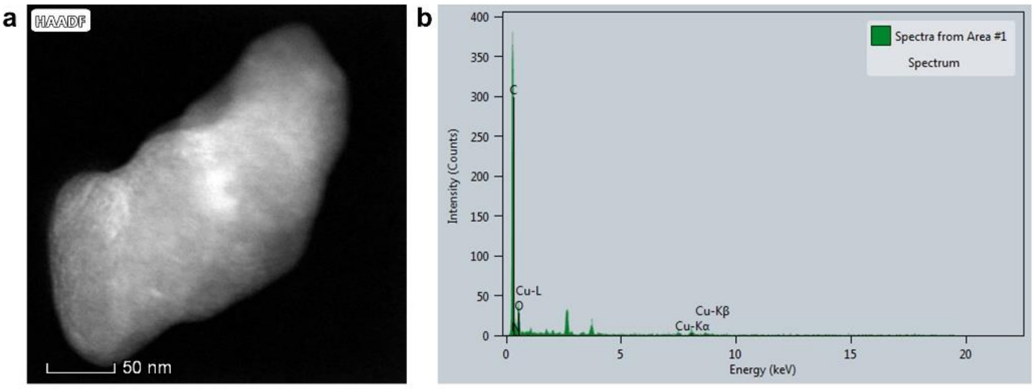

Fig. S4 The original HAADF-STEM image (**a**) and EDS spectra (**b**) for Fig. 1d

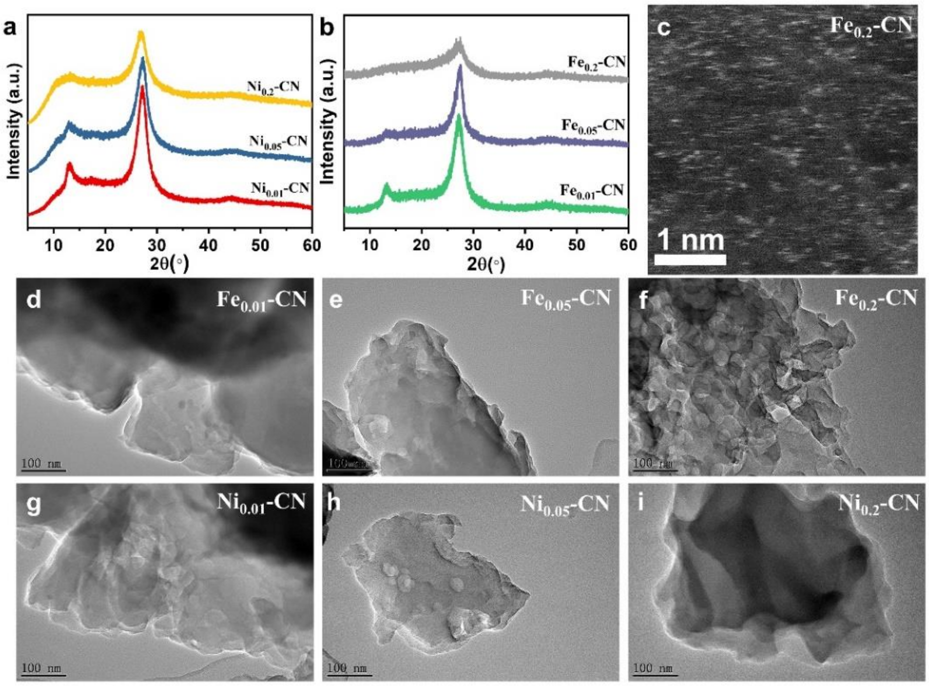

Fig. S5 The characterization of Ni and Fe doped g-C_3N_4 materials. (**a**) XRD patterns of Ni_x-CN materials. (**b**) XRD patterns of Fe_x-CN materials. (**c**) Aberration-corrected HAADF-STEM images of $Fe_{0.2}$-CN. (**d**) TEM image of $Fe_{0.01}$-CN. (**e**) TEM image of $Fe_{0.05}$-CN. (**f**) TEM image of $Fe_{0.2}$-CN. (**g**) TEM image of $Ni_{0.01}$-CN. (**h**) TEM image of $Ni_{0.05}$-CN. (**i**) TEM image of $Ni_{0.2}$-CN

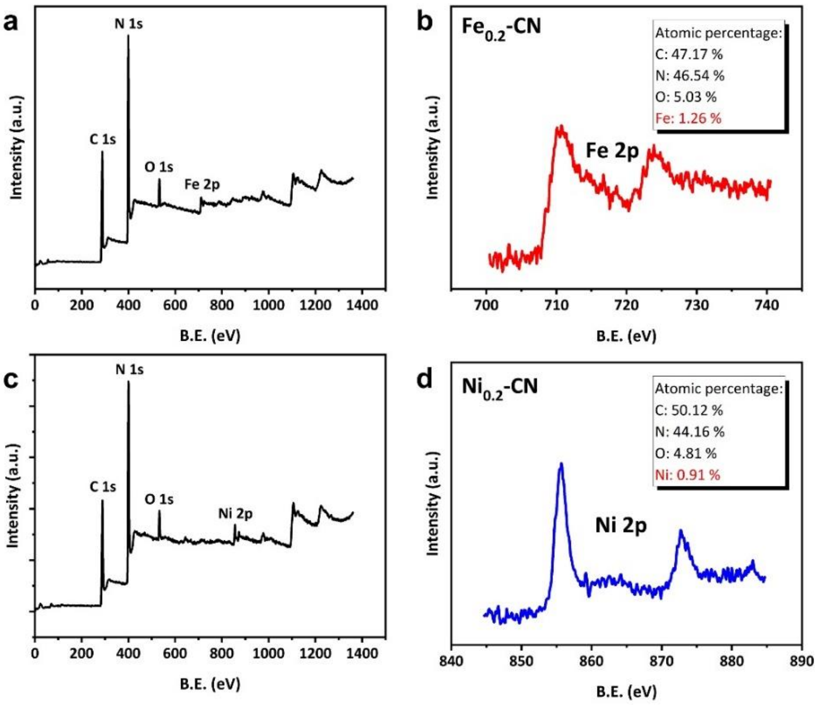

Fig. S6 (**a**) XPS survey spectra of $Fe_{0.2}$-CN. (**b**) Fe 2p spectra of $Fe_{0.2}$-CN. (**c**) XPS survey spectra of $Ni_{0.2}$-CN. (**d**) Ni 2p spectra of $Ni_{0.2}$-CN. Inserts in (**b**) and (**d**) depict the atomic ratio of $Fe_{0.2}$-CN and $Ni_{0.2}$-CN, showing a slightly lower metal content than $Cu_{0.2}$-CN (Cu: 1.75 at%)

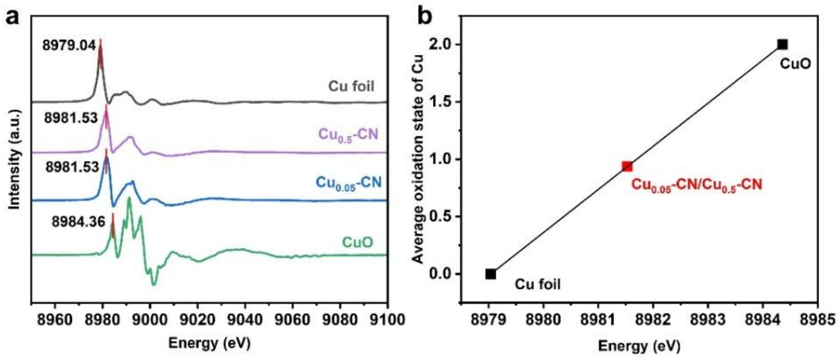

Fig. S7 (**a**) The first derivatives of the Cu K edge XANES spectra of the Cu_x-CN and reference samples in Figure 2d. (**b**) The estimated average oxidation state of Cu in $Cu_{0.05}$-CN and $Cu_{0.5}$-CN catalysts

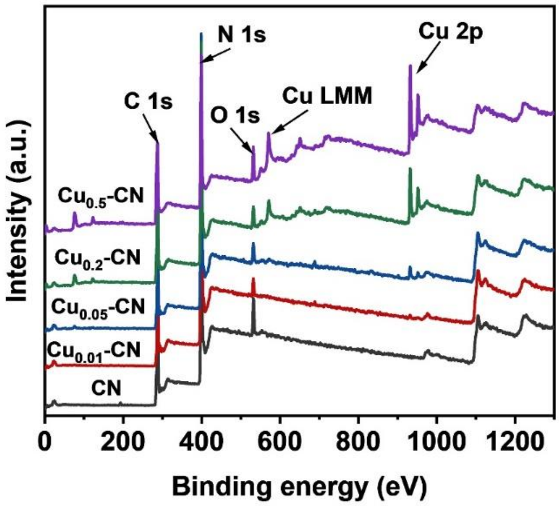

Fig. S8 XPS spectra of different catalysts

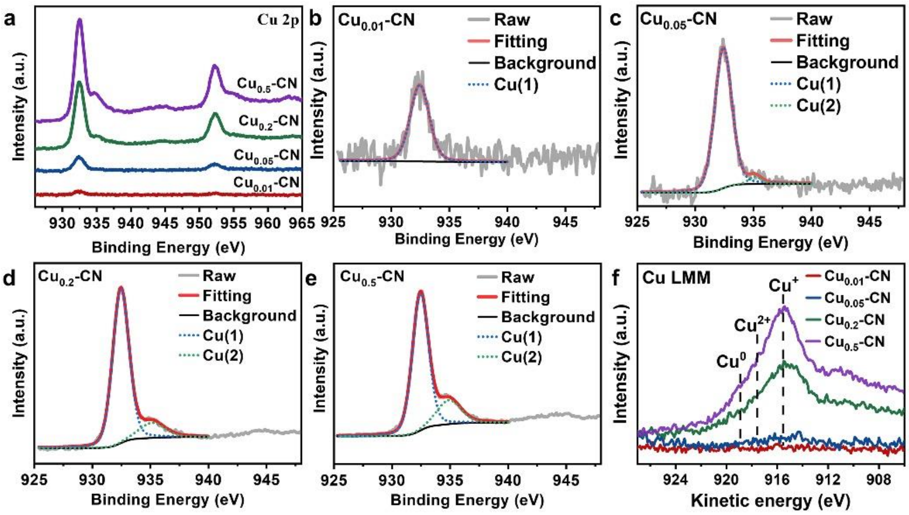

Fig. S9 (**a**) Cu 2p spectra of different Cu_x-CN catalysts. Peak fitting of Cu 2p spectrum of (**b**) $Cu_{0.01}$-CN, (**c**) $Cu_{0.05}$-CN, (**d**) $Cu_{0.2}$-CN, and (**e**) $Cu_{0.5}$-CN catalyst. (**f**) Cu LMM spectra of Cu_x-CN catalysts

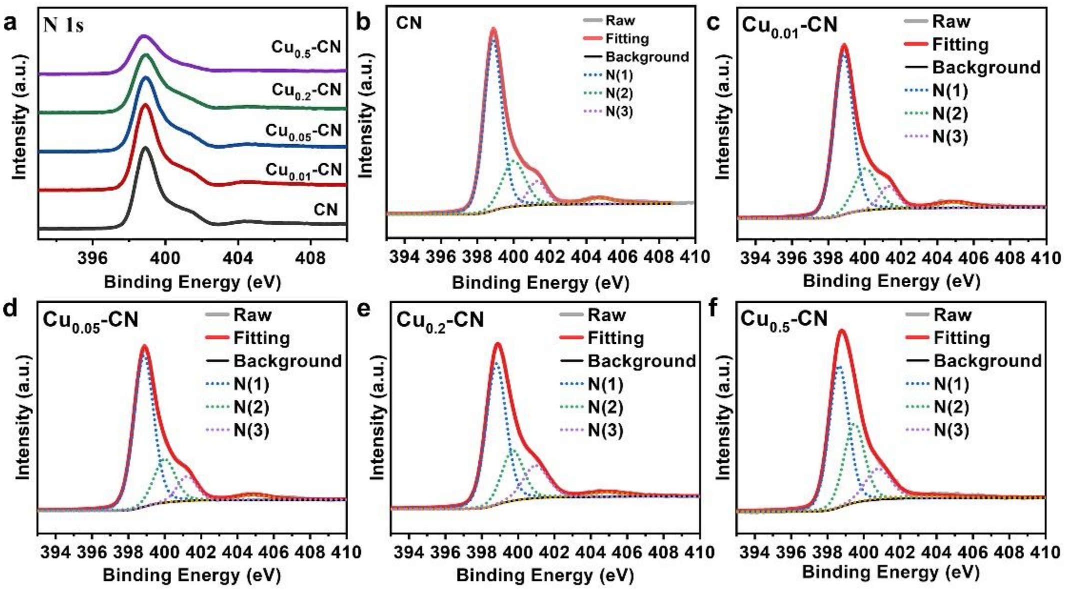

Fig. S10 (**a**) N 1s spectra of different catalysts. Peak fitting of N 1s spectrum of (**b**) CN, (**c**) $Cu_{0.01}$-CN, (**d**) $Cu_{0.05}$-CN, (**e**) $Cu_{0.2}$-CN, and (**f**) $Cu_{0.5}$-CN catalyst

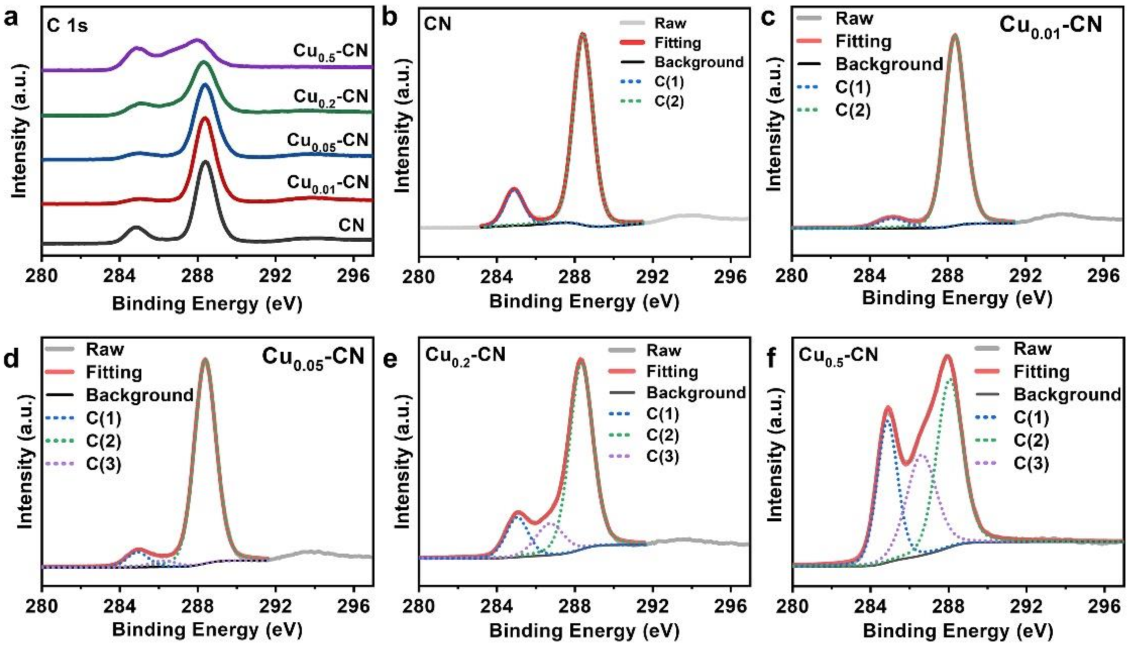

Fig. S11 (**a**) C 1s spectra of different catalysts. Peak fitting of C 1s spectrum of (**b**) CN, (**c**) $Cu_{0.01}$-CN, (**d**) $Cu_{0.05}$-CN, (**e**) $Cu_{0.2}$-CN, and (**f**) $Cu_{0.5}$-CN catalyst

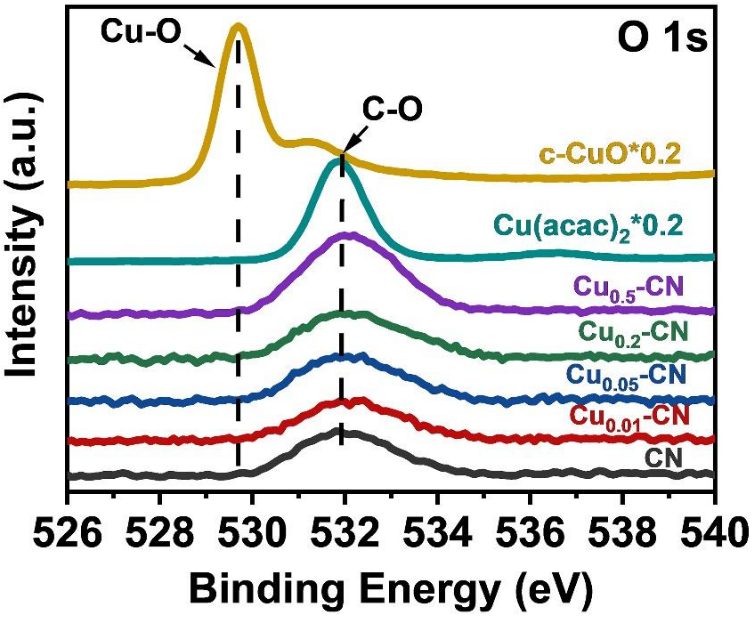

Fig. S12 The C 1s spectra of commercial CuO nanoparticle (c-CuO), Cu(acac)$_2$, and different Cu$_x$-CN catalysts

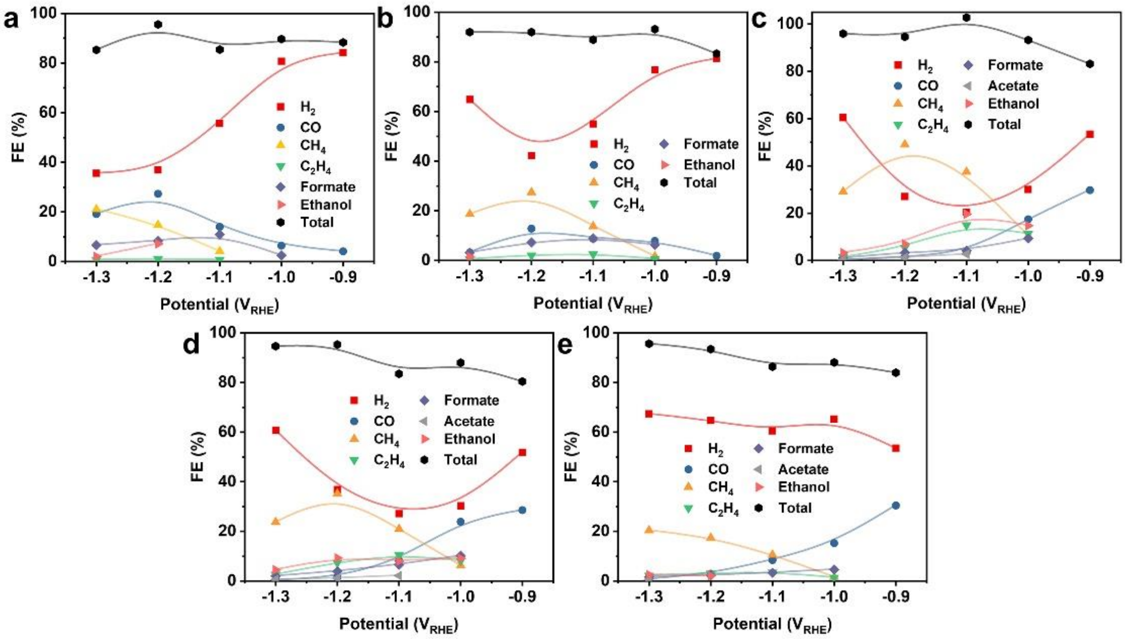

Fig. S13 The product distribution on different catalysts. (**a**) CN. (**b**) Cu$_{0.01}$-CN. (**c**) Cu$_{0.05}$-CN. (**d**) Cu$_{0.2}$-CN. (**e**) Cu$_{0.5}$-CN

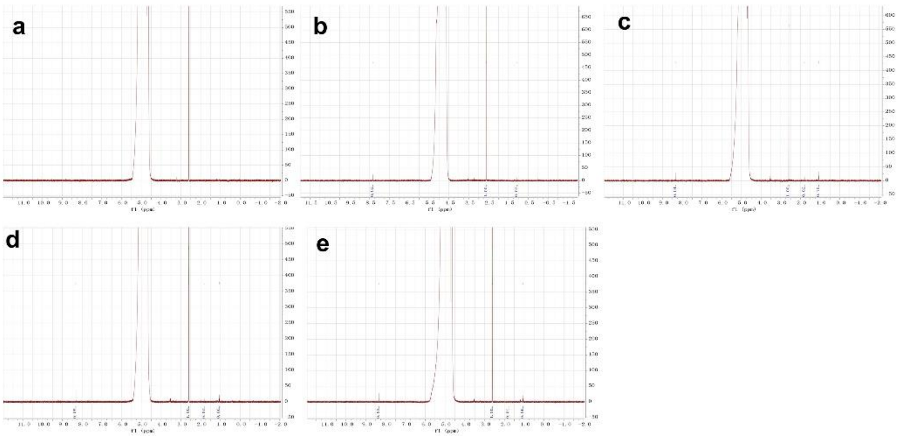

Fig. S14 The ^{1}H NMR spectra of the electrolyte for $Cu_{0.05}$-CN catalysts at different potentials. (**a**) At -0.9 V_{RHE}. (**b**) At -1.0 V_{RHE}. (**c**) At -1.1 V_{RHE}. (**d**) At -1.2 V_{RHE}. (**e**) At -1.3 V_{RHE}

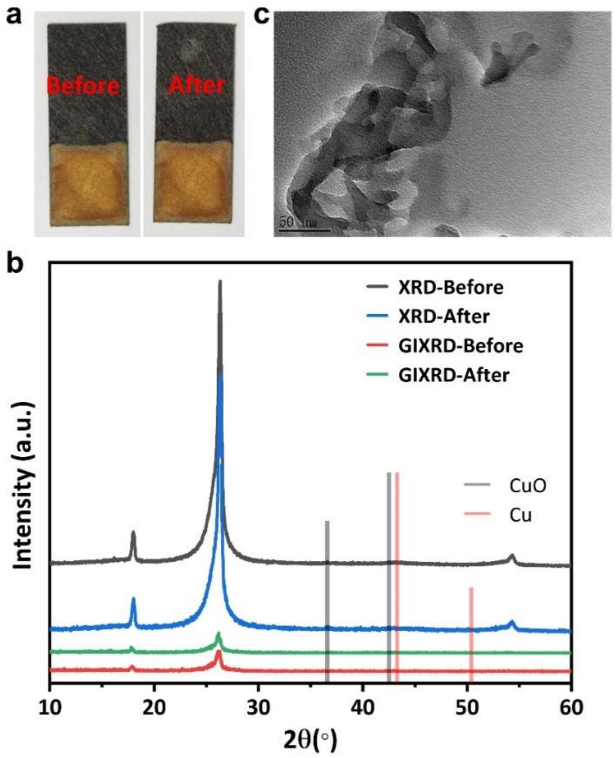

Fig. S15 To determine whether the Cu atoms would agglomerate after CO_2RR electrolysis, a working electrode prepared by coating 2 mg of the $Cu_{0.05}$-CN catalyst (without carbon black) on a piece of carbon paper (1 cm^2) is tested under CO_2RR condition at -1.2 V_{RHE} for 2 hours. (**a**) The photographs of the working electrode before and after CO_2RR electrolysis. (**b**) The XRD and grazing incidence X-ray diffraction (GIXRD) of the working electrode before and after CO_2RR electrolysis. The XRD patterns show no crystalline Cu species (CuO or metallic Cu) form after CO_2RR electrolysis. Further, we also perform GIXRD with an incident angle of 1 degree, which is sensitive to the surface catalyst layer on the electrode. It can be seen that the diffraction peaks of the carbon paper substrate are largely decreased. Similarly, there is

still no crystalline copper species can be detected. (**c**) The TEM image of the $Cu_{0.05}$-CN catalyst peeled off from the electrode after CO_2RR electrolysis. Although the TEM image is less clear than that of the as-prepared $Cu_{0.05}$-CN catalyst due to the addition of Nafion binder, the layered structure of the $Cu_{0.05}$-CN catalysts without crystalline Cu species can be observed.

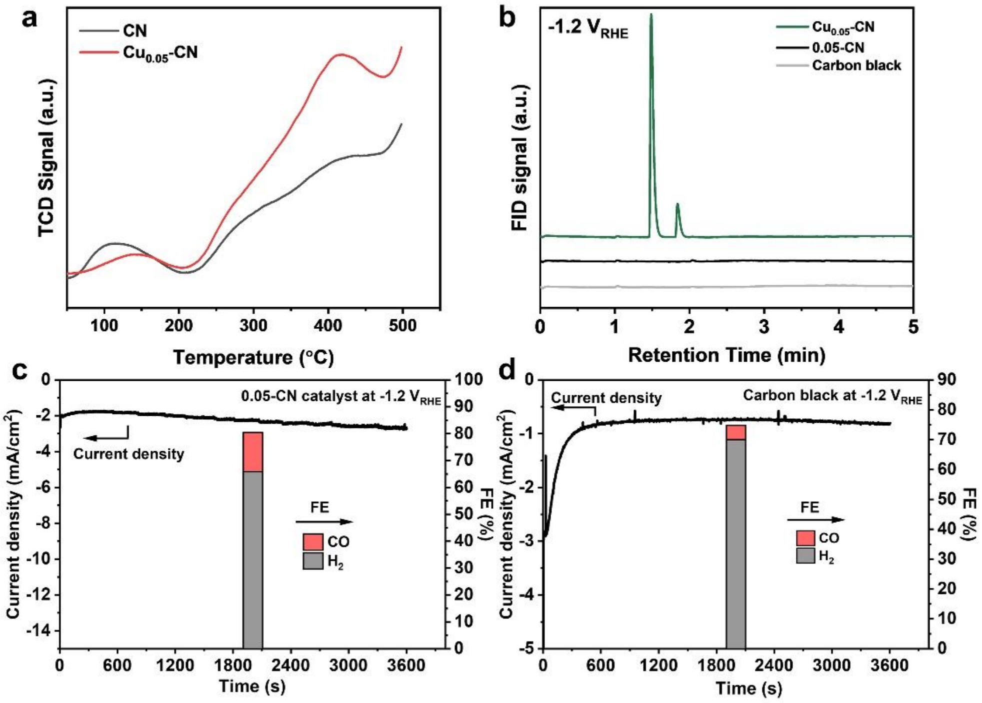

Fig. S16 The CO-TPD measurement and control samples. (**a**) CO-TPD curves of CN and $Cu_{0.05}$-CN catalysts. (**b**) The GC signal of the hydrocarbon products on $Cu_{0.05}$-CN catalyst and control samples, including 0.05-CN and carbon black. (**c**) FEs and current density of 0.05-CN catalyst. (**d**) FEs and current density of carbon black

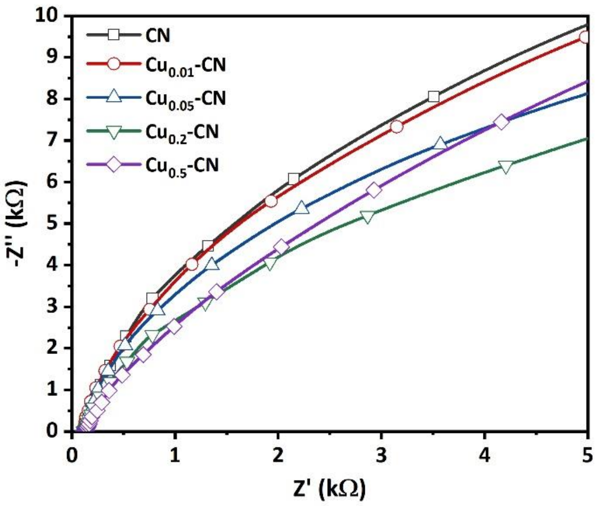

Fig. S17 The Nyquist plots of CN and Cu_x-CN catalysts in CO_2-saturated 0.1 M $KHCO_3$

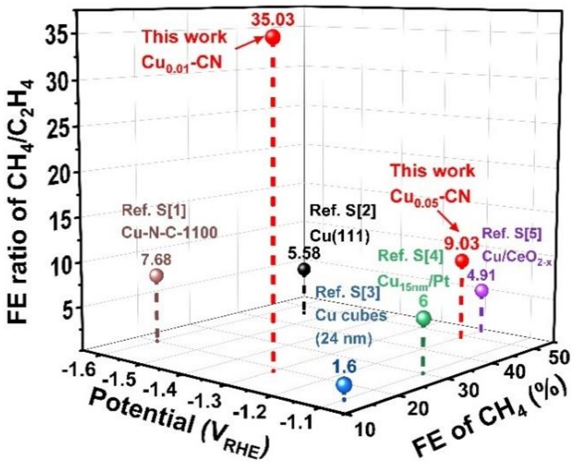

Fig. S18 The comparison of CO_2RR performance and FE ratios of CH_4/C_2H_4 with the reported literatures [S1-S5]

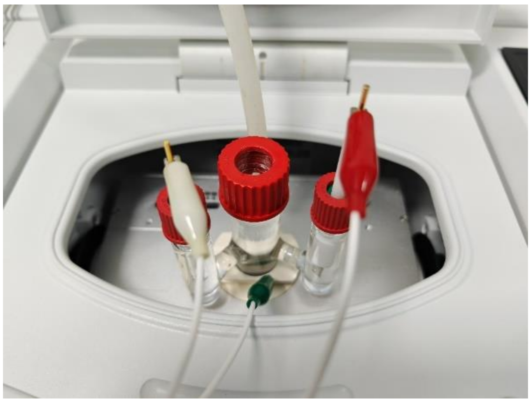

Fig. S19 The electrochemical cell for in situ ATR-FTIR measurement

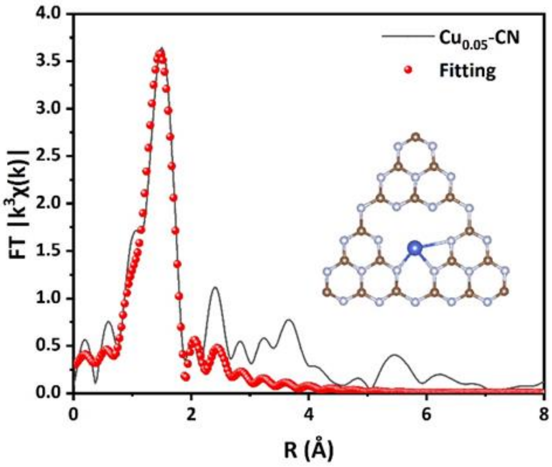

Fig. S20 The fitting curve of k^3-weighted EXAFS spectra in R space of $Cu_{0.05}$-CN, using the Artemis module of IFEFFIT. The corresponding model is shown as insert. The fitting for $Cu_{0.05}$-CN was done on the k^3-weighted EXAFS function $\chi(k)$ data from 2.7 to 11 Å^{-1} in the R-range of 1.0–2.0 Å. The fitting parameters are listed in Table S2

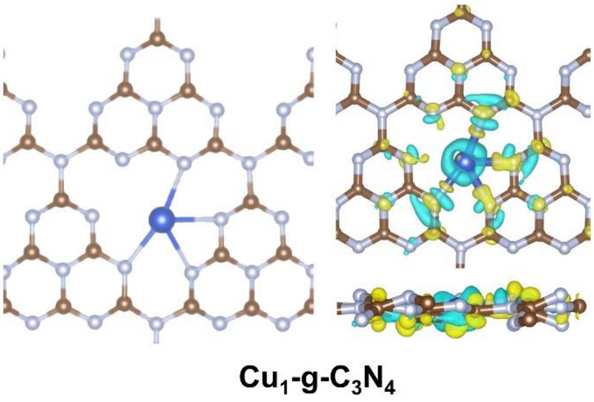

Fig. S21 The optimized structure and the differential charge densities of Cu_1-g-C_3N_4. Yellow represents electron accumulation and cyan denotes electron depletion. It is clear that the electrons transfer from Cu atoms to the coordinated N atoms

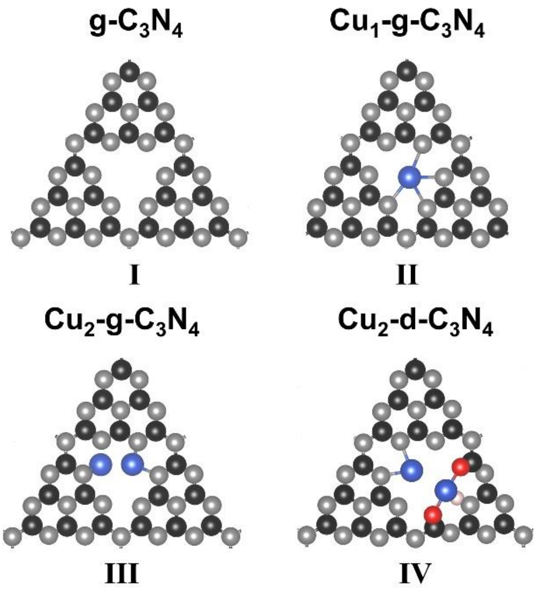

Fig. S22 The models of pristine g-C_3N_4 (I), Cu_1-g-C_3N_4 (II), Cu_2-g-C_3N_4 (III), and Cu_2-d-C_3N_4 (IV). Based on the experimental results, four models were first built, namely the pristine carbon nitride (g-C_3N_4), one Cu atom incorporated in the nitrogen cavities of g-C_3N_4 (Cu_1-g-C_3N_4), two Cu atoms incorporated in the nitrogen cavities of g-C_3N_4 (Cu_2-g-C_3N_4), and the damaged g-C_3N_4 framework hosted two adjacent atomic Cu sites with different coordination structures (Cu_2-d-C_3N_4)

Note: Based on the structural and compositional characterizations, the C-N heterocycles of g-C_3N_4 are partially damaged and the defects increase in the Cu_x-CN catalysts with high Cu loads, including the $Cu_{0.2}$-CN and $Cu_{0.5}$-CN catalysts. Meanwhile, the increasing O and Cu content and the emergance of Cu^{2+} species indicate the formation of Cu sites with O-coordination in the $Cu_{0.2}$-CN and $Cu_{0.5}$-CN catalysts. Therefore, the Cu_2-d-C_3N_4 structure is proposed to investigate the CO_2RR on the $Cu_{0.2}$-CN and $Cu_{0.5}$-CN catalysts.

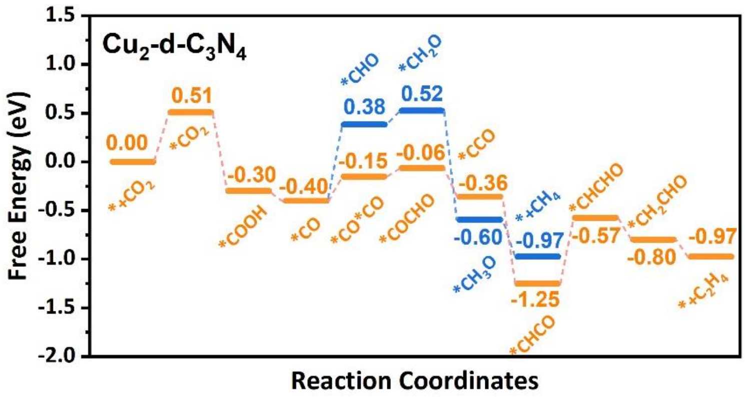

Fig. S23 The free energy diagram of CO_2 to CH_4 and C_2H_4 on Cu_2-d-C_3N_4

Table S1 The Cu content in CN and Cu_x-CN catalysts determined by ICP-AES

Catalysts	CN	$Cu_{0.01}$-CN	$Cu_{0.05}$-CN	$Cu_{0.2}$-CN	$Cu_{0.5}$-CN	0.05-CN
Cu (wt.%)	/	0.210 %	0.954 %	2.554 %	3.811 %	0.003 %

Table S2 EXAFS fitting parameters for $Cu_{0.05}$-CN

Sample	Path	N	R (Å)	σ^2 (10^{-3} Å^2)	ΔE_0 （eV）	R factor
$Cu_{0.05}$-CN	Cu-N	3.33	1.92	8.05	1.1	0.016

Supplementary References

[S1] A. Guan, Z. Chen, Y. Quan, C. Peng, Z. Wang, T.-K. Sham, C. Yang, Y. Ji, L. Qian, X. Xu, G. Zheng. Boosting CO_2 electroreduction to CH_4 via tuning neighboring single-copper sites. ACS Energy Lett. **5**(4), 1044-1053 (2020). https://doi.org/10.1021/acsenergylett.0c00018

[S2] Y. Hori, I. Takahashi, O. Koga, N. Hoshi. Electrochemical reduction of carbon dioxide at various series of copper single crystal electrodes. J. Mol. Catal. A: Chem. **199**(1), 39-47 (2003). https://doi.org/10.1016/S1381-1169(03)00016-5

[S3] A. Loiudice, P. Lobaccaro, E. A. Kamali, T. Thao, B. H. Huang, J. W. Ager, R. Buonsanti. Tailoring copper nanocrystals towards C_2 products in electrochemical CO_2 reduction. Angew. Chem. Int. Ed. **55**(19), 5789-5792 (2016). https://doi.org/10.1002/anie.201601582

[S4] R. Reske, M. Duca, M. Oezaslan, K. J. P. Schouten, M. T. M. Koper, P. Strasser. Controlling catalytic selectivities during CO_2 electroreduction on thin cu metal overlayers. J. Phys. Chem. Lett. **4**(15), 2410-2413 (2013).

https://doi.org/10.1021/jz401087q

[S5] S. B. Varandili, J. Huang, E. Oveisi, G. L. De Gregorio, M. Mensi, M. Strach, J. Vavra, C. Gadiyar, A. Bhowmik, R. Buonsanti. Synthesis of Cu/CeO_{2-x} nanocrystalline heterodimers with interfacial active sites to promote co_2 electroreduction. ACS Catal. **9**(6), 5035-5046 (2019). https://doi.org/10.1021/acscatal.9b00010
